# TLR2 mediates cell cycle re-entry and survival of neonatal cardiomyocytes in response to injury

**DOI:** 10.1186/s12964-026-03147-w

**Published:** 2026-08-14

**Authors:** Julia Nicke, Adrian Goldspink, Malte Menn, Bernd K. Fleischmann, Mona Malek Mohammadi

**Affiliations:** Institute of Physiology I, Medical Faculty, University of Bonn, Venusberg-Campus 1, Building 76, Bonn, D-53127 Germany

## Abstract

Unlike adult hearts, neonatal hearts can regenerate and adapt to stress. To identify both cardioprotective and regenerative mechanisms, we compared activated genes and related signaling pathways in the neonatal mouse heart after myocardial infarction (MI) and after exposure to pressure overload using a neonatal model of transverse aortic constriction (nTAC) at postnatal day 1 (P1). We identified three immune-related genes—Ccl4, S100a8, and C1qa—of high interest, as they encode secreted factors, are highly expressed in the neonatal mouse heart in both injury types, and their receptors are expressed on neonatal cardiomyocytes (nCM) and cardiac endothelial cells (EC). We investigated their effects on primary mouse EC and nCM in vitro. Our study found that the combination of these secreted factors (Pool3) enhances EC and nCM cell cycle re-entry and reduces the rate of apoptosis. Combined in vivo and in vitro transcriptome analyses revealed that Toll-like receptor 2 (TLR2) activation in nCM induces a shared pro-survival and cell cycle re-entry-associated signaling, including upregulation of Bcl2 and Birc5, respectively. The direct and pivotal role of TLR2 in enhancing nCM cell cycle re-entry and survival was further confirmed using TLR2 knockout (KO) nCM, a TLR2 inhibitor, and a TLR2 agonist (zymosan). Moreover, the importance of TLR2 in mediating the adaptive response to pressure overload in neonatal mice was confirmed in TLR2 KO mice, which failed to adapt to pressure overload and exhibited high mortality together with maladaptive cardiac remodeling as early as 7 days post-surgery. Collectively, these findings demonstrate that TLR2 is essential for mediating nCM cell cycle re-entry, survival, and adaptive/regenerative response to injury.

**Graphical Abstract:**

Schematic image of immune-cardiomyocyte crosstalk via TLR2 during cardiac injury in neonatal mice. TLR2 activation in response to secreted immune cytokines such as CCL4, C1QA, and S100A8 during cardiac injury promotes nCM survival and cell cycle re-entry via upregulation of Bcl2 and Birc5, respectively

**Figure:**
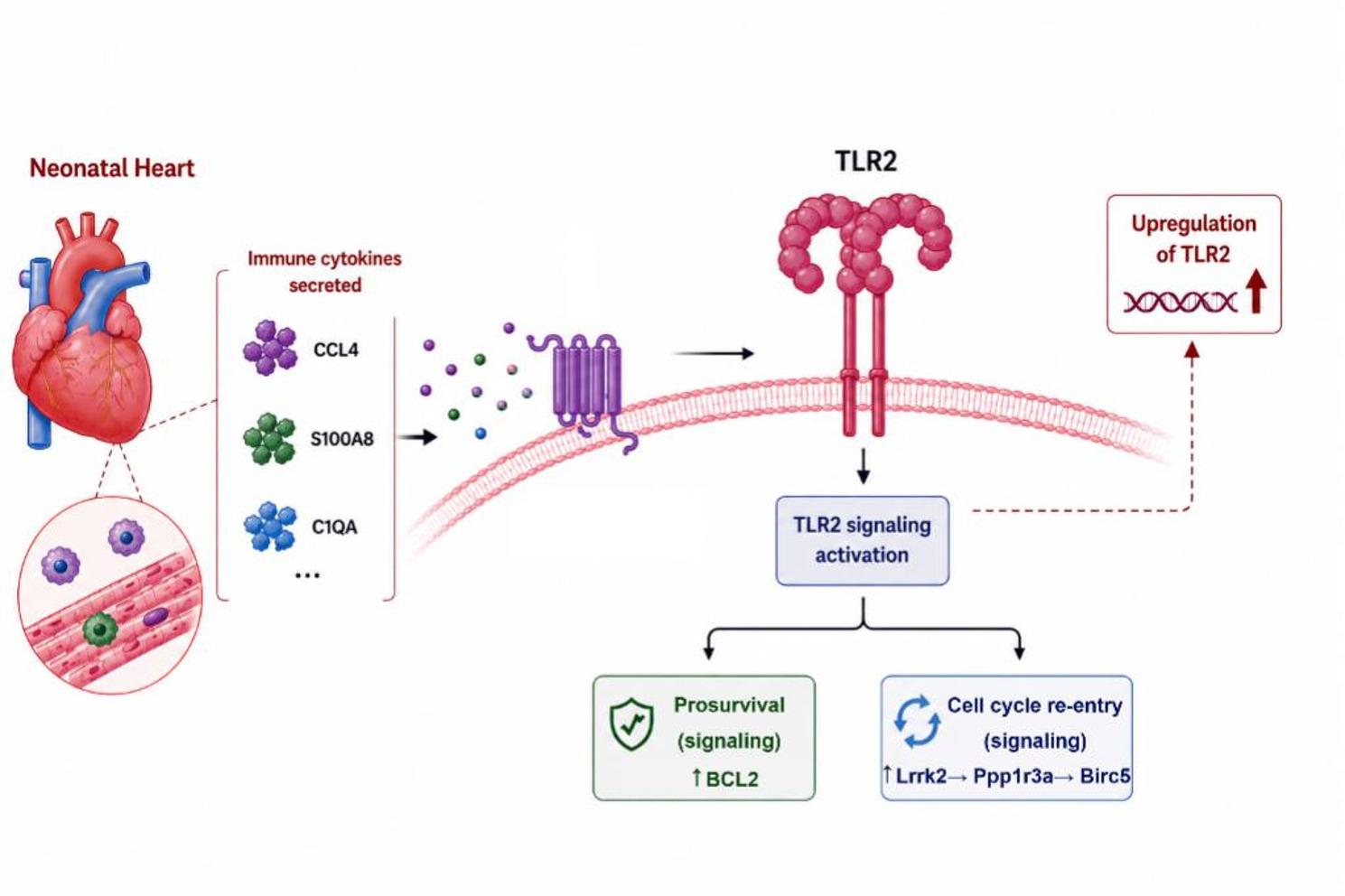

**Supplementary Information:**

The online version contains supplementary material available at 10.1186/s12964-026-03147-w.

## Introduction

Cardiovascular diseases (CVD) are the leading cause of death worldwide, partly due to the limited regenerative capacity of the adult heart, which results from a low cardiomyocyte (CM) renewal rate [1]. In contrast, neonatal mice at postnatal day 1 (P1) can regenerate the heart after focal injuries [2–4] and adapt to pressure overload [5, 6]. Studies have shown that following P1 myocardial infarction (MI), there is a transient activation of pro-regenerative genes in the heart, driving angiogenesis and neonatal (n)CM proliferation [7]. This promotes regeneration of the injured area before gene expression returns to baseline (uninjured heart) by three days post-surgery (dps), at which point regeneration is lost [5, 7]. However, following pressure overload induced by neonatal transverse aortic constriction (nTAC), pro-regenerative genes remain active up to 14 dps, extending the regenerative phase and preventing heart failure [5]. This is particularly remarkable, as by 14 dps the heart has already lost its regenerative capacity, and pressure-overloaded hearts typically begin to decompensate, unlike after MI, which triggers immediate failure. This sustained activation promotes nCM proliferation and angiogenesis, leading to heart enlargement through hyperplasia, while maintaining cardiac function and preventing fibrosis or hypertrophy [5]. However, it remains unclear whether the mechanisms driving cardiac regeneration after MI also protect the heart from pressure overload. Clarifying the similarities and differences between these regenerative processes may uncover new therapeutic strategies to enhance the adult heart’s regenerative capacity.

In this study, we first aimed to identify conserved genes and signaling pathways shared between MI and nTAC, with a particular focus on genes that remain active for extended periods, which could play a crucial role in maintaining the heart’s regenerative capacity. To achieve this, we compared upregulated genes in the heart at 1 dps P1 MI and 14 dps P1 nTAC. To enhance therapeutic relevance, we focused on genes encoding secreted factors that have receptors on nCM and cardiac endothelial cells (EC) but have unknown effects on these cells. This analysis highlighted genes encoding secreted factors, including S100a8, Ccl4, and C1qa. S100A8, also known as calgranulin A, is mainly secreted by neutrophils and monocytes and is linked to inflammation [8, 9]. S100A8 binds to Toll-like receptor 4 (TLR4) and has complex effects that depend on dose, context, and cell type [10–15]. CCL4, a chemokine, is linked to cancer prognosis and progression and has been shown to increase VEGF-C expression and promote lymphangiogenesis [16]. C1q, which includes C1qA, C1qB, and C1qC, is part of the complement system and can promote tumour growth and metastasis [17, 18]. However, the effects of these three factors on nCM and EC have not been thoroughly investigated, nor have their roles in nCM and EC biology during heart regeneration been elucidated.

Treating mouse primary nCM and EC with a combination of all three proteins (Pool3) revealed pro-survival effects and enhanced cell cycle re-entry activity. Mechanistically, we identified Toll-like receptor 2 (TLR2) on nCM that drives downstream pro-survival and cell cycle re-entry-associated signaling pathways in response to Pool3 treatment. Using TLR2 knock-out (KO) nCM, a TLR2 inhibitor, and a TLR2 agonist, we confirmed the critical role of TLR2 in enhancing nCM survival and cell cycle re-entry. Moreover, the crucial role of TLR2 in mediating the adaptive response to pressure overload in neonatal mice was validated in TLR2 KO mice, which failed to adapt to pressure overload and exhibited high mortality together with maladaptive cardiac remodeling as early as 7 dps, characterised by nCM hypertrophy and impaired ability to induce angiogenesis and nCM cell cycle re-entry.

## Results

### Pressure overload at P1 prolongs activation of pro-regenerative and protective genes, reflecting the early regenerative response to MI

**Fig. 1:**
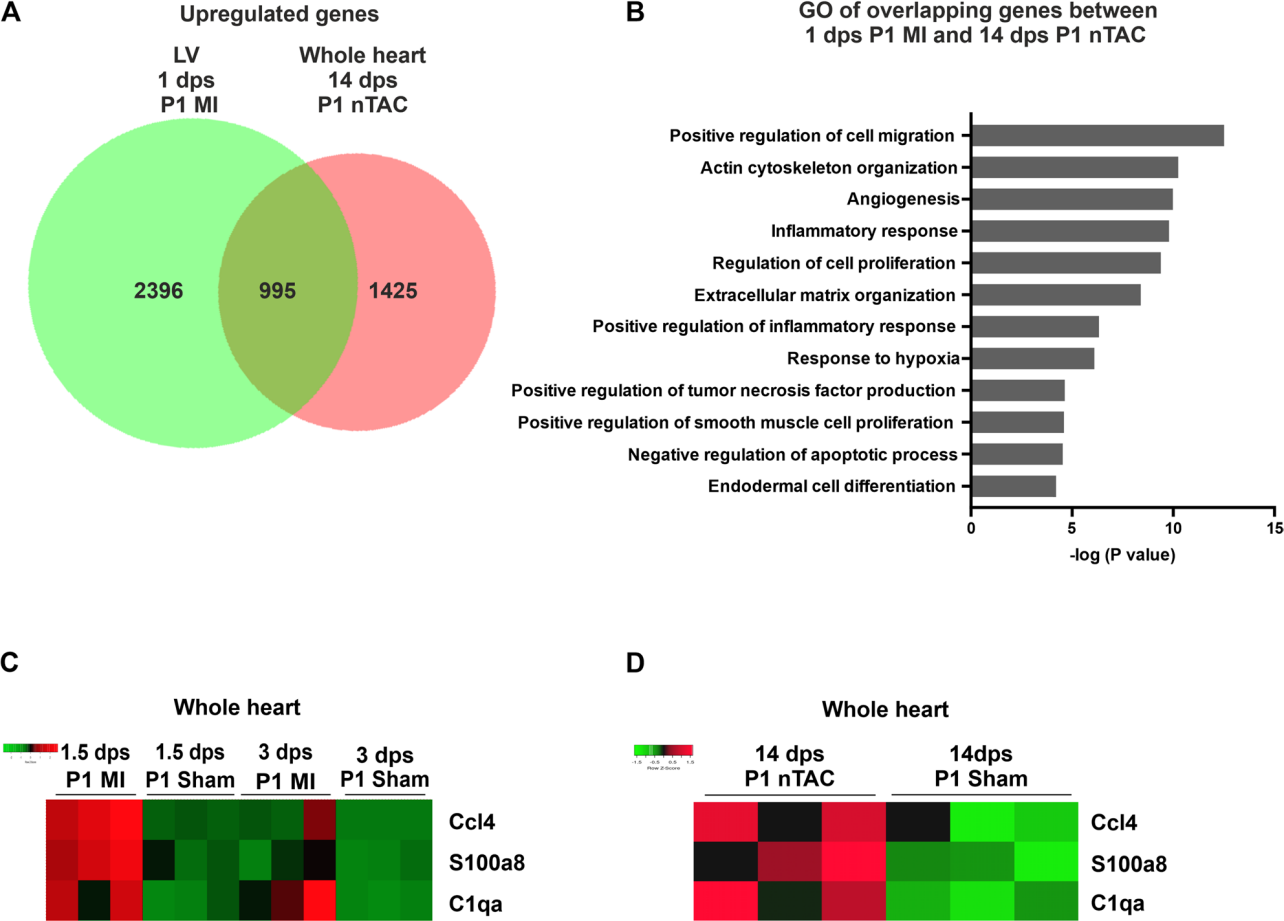
Pressure overload maintains the overexpression of pro-regenerative genes that are normally transiently activated after MI. **A** Venn diagram showing similarities in overexpressed genes at 1 dps P1 MI with those at 14 dps P1 nTAC. **B** Gene Ontology (GO) analysis of overlapping genes at 1 dps P1 MI compared to 14 dps P1 nTAC. **C** Heatmap showing gene expression of Ccl4, S100a8, and C1qa at 1.5 dps and 3 dps P1 MI and sham hearts, obtained from database [7]. **D** Heatmap showing gene expression of Ccl4, S100a8, and C1qa in 14 dps P1 nTAC and sham hearts [5]

First, we aimed to identify conserved cardioprotective signaling that is activated shortly after P1 MI and maintained under conditions of prolonged pressure overload induced by nTAC. For this purpose, we performed a comparative analysis of upregulated genes in the left ventricle (LV) at 1 dps P1 MI and at 14 dps P1 nTAC whole hearts using our published sequencing data [5, 19]. Our analysis revealed that nearly 41% (995 genes) of the genes overexpressed in the heart after 14 days of pressure overload are also upregulated in the LV shortly (1 dps) after P1 MI (Fig. 1A). Gene ontology (GO) analysis of these overlapping genes revealed biological processes including positive regulation of cell migration, angiogenesis, inflammatory response, cell proliferation, and negative regulation of apoptosis (Fig. 1B). This suggested that conserved cardioprotective mechanisms, such as anti-apoptotic, pro-angiogenic, and pro-proliferative processes, exhibit prolonged activation in response to pressure overload in the neonatal mouse heart.

We next assessed the molecular function of these overlapping genes bioinformatically and found that many encode ligands that are protein- or calcium-binding proteins (Supplementary Fig. 1A). Cellular component analysis revealed that 142 genes encode secreted factors, such as S100a8 and C1qa (Supplementary Fig. 1B). Further investigations showed that Ccl4 is typically co-expressed with C1qa, and this is uniquely upregulated during the regenerative response of the P1 MI but not in P3 MI, a time point lacking cardiac regeneration [19]. Assessing another published sequencing dataset covering various time points after P1 MI [7] revealed that Ccl4, S100a8, and C1qa are highly expressed in the whole heart at 1.5 dps in P1 MI, but their expression declines shortly (3 dps) thereafter (Fig. 1C). However, the nTAC transcriptomic dataset showed that S100a8 and C1qa are upregulated with a similar trend for Ccl4 up to 14 days after pressure overload (Fig. 1D).

**Fig. 2:**
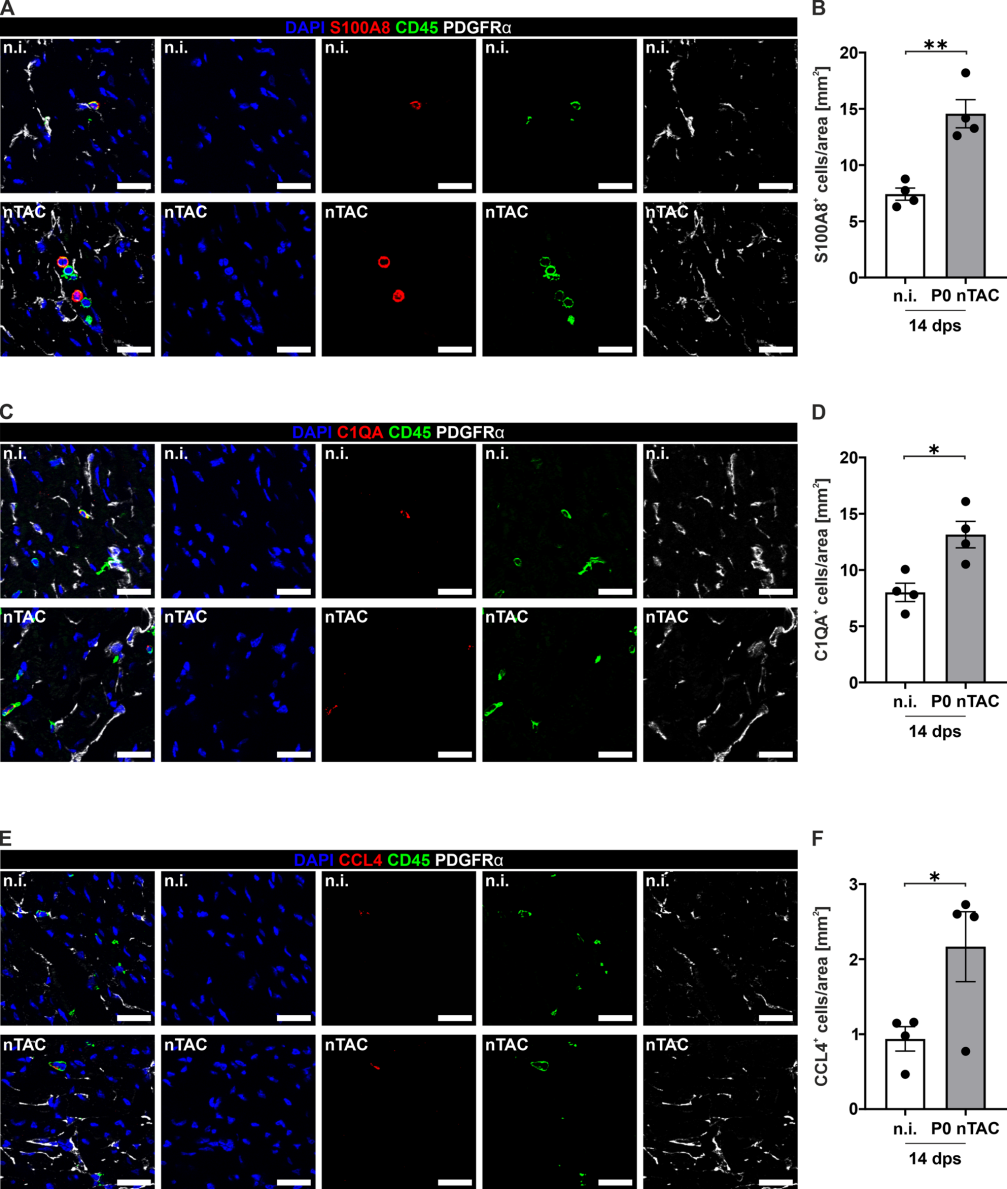
Pressure overload increases the number of CCL4, C1QA, and S100A8-expressing cells in the heart. **A-F** Immunostaining of heart sections at P14 in non-injured (n.i.) control and 14 dps P0 nTAC for CD45, PDGFRα, DAPI, and S100A8 (A), C1QA (C), CCL4 (E), and quantification of S100A8^+^ cells (B), C1QA^+^ cells (D), and CCL4^+^ cells (F) per area. Scale bar = 20 μm, (*p < 0.05; **p < 0.01; unpaired t-test)

Immunostaining of heart sections for CCL4, C1QA, and S100A8, together with CD45 and PDGFRα, confirmed an increased number of positive cells for all 3 proteins following P0 nTAC at 14 dps, predominantly within the CD45⁺ population (Fig. 2A–F). Notably, C1QA⁺ cells were already significantly increased in P0 nTAC at 3 dps, whereas no significant changes were detected for CCL4 or S100A8 compared with controls (Supplementary Fig. 1C–H).

Since S100a8, C1qa, and Ccl4 encode secreted factors, we first identified their receptors using an online database [20] and confirmed the interactions using STRING analysis (Supplementary Fig. 2A-B). Transcriptome analysis of nCM and EC using a public database [21] revealed that the genes related to their receptors, such as Ackr2, Cc3, Ccr8, Tlr4, Cd93, Cr1l, Cspg4, and Corin, are expressed in both nCM and EC, indicating their potential interaction and reciprocal crosstalk (Supplementary Fig. 2C-D). Further analysis of the database containing transcriptomes of cardiac cell types [21] revealed that Ccl4, S100a8, and C1qa are highly expressed in both cardiac fibroblasts (FB) and immune cells at 2 dps P1 MI, indicating that both cell types could contribute to the secretion of these factors (Supplementary Fig. 2E-F). However, comparative analysis of expression levels between FB and immune cells identified immune cells as the predominant source (Supplementary Fig. 2G-I), underscoring our immunostaining data (Fig. 2A, C, and E). Thus, we demonstrated that Ccl4, S100a8, and C1qa are upregulated in the neonatal heart during regeneration, with nCM and EC as potential target cells.

We therefore tested the biological effects of these proteins on nCM and EC by applying each factor individually or in combination (Pool3). After testing various concentrations and durations of the proteins on nCM—which are particularly challenging due to their limited capacity for cell cycle re-entry, and cytokinesis—we identified optimal concentrations that promote cytokinesis (Aurora B^+^ nCM) after 48 hours (h) (Supplementary Fig. 3A-F). Based on these findings, we initiated experiments using CCL4 at 100 ng/ml, S100A8 at 50 ng/ml, and C1QA at 200 ng/ml.

We first tested the effects of the three proteins on human umbilical vein endothelial cells (HUVEC), as previous studies indicated that each, namely CCL4, S100A8, and C1QA, had positive effects on HUVEC [10, 22, 23]. We found that treatment of HUVEC with CCL4, S100A8, C1QA, or Pool3 did not influence their proliferation rate (Supplementary Fig. 3G-H). However, the apoptosis rate assessed by cleaved Caspase 3 (cCasp3) immunostaining was reduced (1.3-fold) when HUVEC were treated with Pool3 (Supplementary Fig. 4A-B), but not with the individual factors. The scratch assay revealed improved migration ability of HUVEC in vitro upon treatment with Pool3 (Supplementary Fig. 4C-D). Additionally, the tube formation assay showed an increased number of branching points and closed tubes with Pool3 in HUVEC (Supplementary Fig. 4E-G). These findings suggested pro-survival and pro-angiogenic effects of Pool3 factors on HUVEC.

### Pool3 enhances nCM and EC survival and cell cycle re-entry in co-culture

**Fig. 3:**
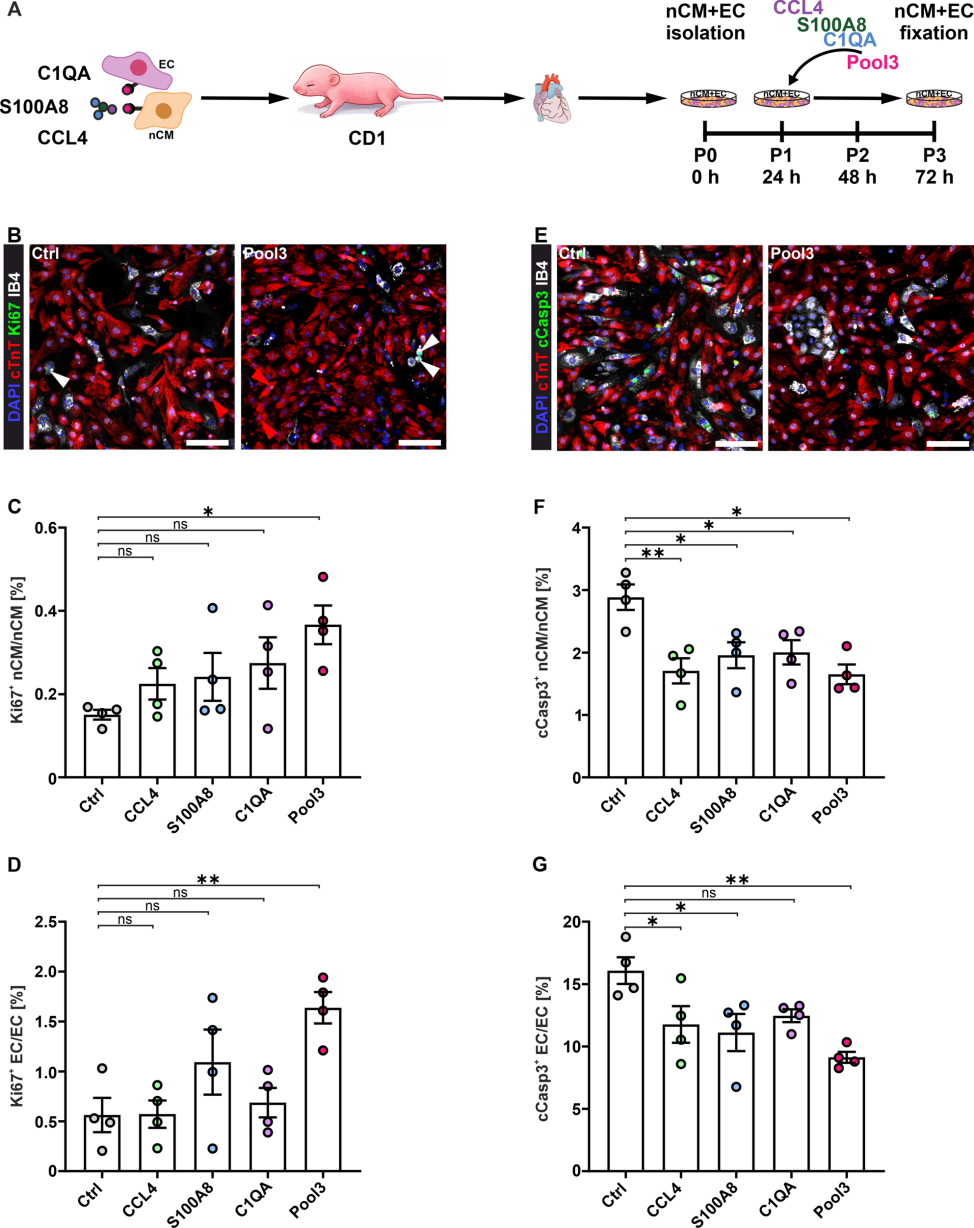
Pool3 enhances cell cycle activity and reduces the apoptosis rate of nCM and EC in vitro. **A** Schematic image of the experiment showing co-isolation and co-culture of nCM and EC and treatment with the proteins. **B-D** Immunofluorescence images and quantification of Ki67^+^ nCM (C, red arrowheads in B) and EC (D, white arrowheads in B) in control (Ctrl) and Pool3-treated nCM. **E-G** Immunofluorescence images and quantification of cCasp3^+^ nCM (F) and EC (G). Scale bar = 100 μm, (*p < 0.05; **p < 0.01; ns: not significant; one-way ANOVA with Šídák’s multiple comparisons test)

Since our primary goal is to study the effects of these proteins in the heart, we investigated whether they exert similar beneficial effects on murine primary nCM and EC. To this end, we co-isolated nCM and EC from P0-P1 mouse hearts and treated them with individual proteins or with Pool3 (Fig. 3A). Quantification of Ki67 showed that Pool3, but not the individual proteins, enhanced cell cycle activity of both nCM (2.5-fold) and EC (3.7-fold) (Fig. 3B-D). Moreover, each protein individually and Pool3, to a greater extent, exhibited a clear pro-survival effect on both nCM and EC, as evidenced by reduced cCasp3^+^ nCM (1.7-fold) and EC (1.7-fold) compared to controls (Fig. 3E-G).

### Pool3 directly enhances nCM cell cycle re-entry and survival rate

**Fig. 4:**
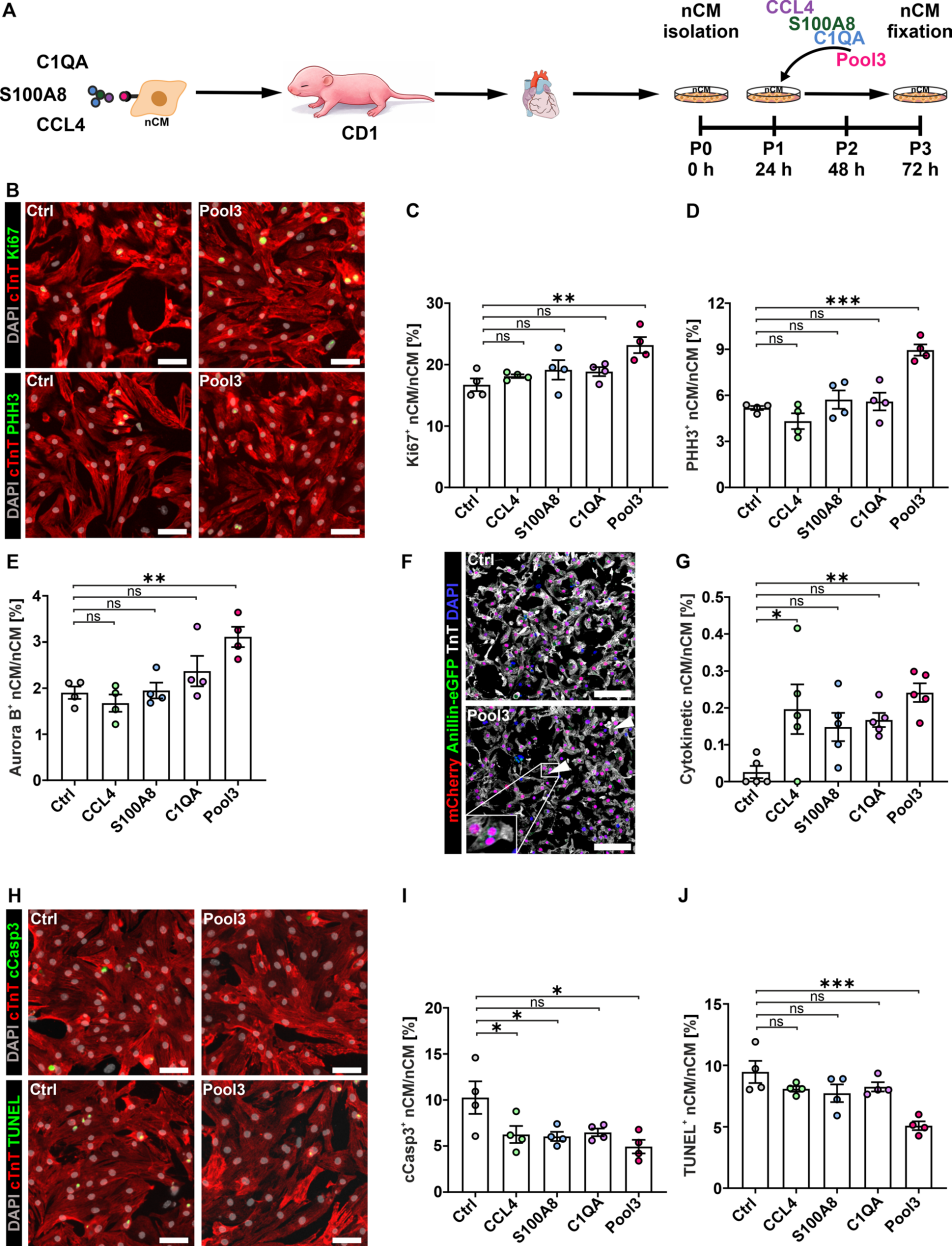
Pool3 enhances nCM cell cycle activity and survival in vitro. **A** Schematic image of the experiment showing isolation of primary mouse nCM from P0-P1 hearts and in vitro treatment with CCL4, S100A8, C1QA, or Pool3. **B-D** Immunofluorescence images of Ki67 and PHH3 staining in nCM in Ctrl and Pool3-treated nCM (B) and quantification of Ki67^+^ (C) and PHH3^+^ nCM (D). **E** Quantification of Aurora B^+^ nCM. **F-G** Anillin^+^ mid-body shown by arrowhead and enlarged inside the insert, and its quantification (G). **H-J** Immunofluorescence images of cCasp3^+^ and TUNEL^+^ nCM (H) and quantification of cCasp3^+^ (I) and TUNEL^+^ (J) nCM. Scale bar = 50 μm, (*p < 0.05; **p < 0.01; ***p < 0.001; ns: not significant; one-way ANOVA with Šídák’s multiple comparisons test)

To further investigate the direct effects of the proteins on nCM cell cycle re-entry and survival, while excluding potential secondary effects or crosstalk from EC, we isolated primary mouse nCM and treated them with individual proteins or Pool3 (Fig. 4A). Quantification of Ki67^+^, PHH3^+^, and Aurora B^+^ nCM revealed that Pool3 enhances the rates of cell cycle activity (1.4-fold), mitosis (1.7-fold), and cytokinesis (1.7-fold) in nCM, respectively (Fig. 4B-E, Supplementary Fig. 5A). Given the known challenges in distinguishing cytokinesis from binucleation in nCM, we further validated nCM cell cycle re-entry using nCM from double transgenic H2B-mCherry-Anillin-eGFP mice [24–26]. This enabled us to identify nCM at different stages of the cell cycle based on the subcellular localization of the anillin-eGFP signal [24, 26], while the mCherry signal labels nCM nuclei. Double transgenic nCM were treated with individual proteins or Pool3 (Supplementary Fig. 5B). Quantification of cycling nCM, as well as mitosis and binucleation rates, based on the location of anillin signal showed no significant differences between the treated and non-treated groups (Supplementary Fig. 5C-F). However, quantification of the cytokinesis rate of nCM (anillin signal localized centrally between the two nuclei) revealed a significant increase upon treatment of nCM with CCL4 and, more importantly, with Pool3 (Fig. 4F-G), confirming that Pool3 can induce nCM cell cycle re-entry in vitro.

Additionally, we assessed the apoptosis rate of nCM using cCasp3 immunostaining and the TUNEL assay to detect and quantify nCM in the early and late stages of apoptosis, respectively (Fig. 4H). Pool3 treatment significantly reduced the number of cCasp3^+^ and TUNEL^+^ nCM, consistent with reduced nCM apoptosis rates (2 and 1.8 folds, respectively) (Fig. 4H-J, Supplementary Fig. 5G), highlighting the pronounced pro-survival effect of Pool3 on nCM.

### Transcriptome analysis underscores cell cycle re-entry and pro-survival effects of Pool3

**Fig. 5:**
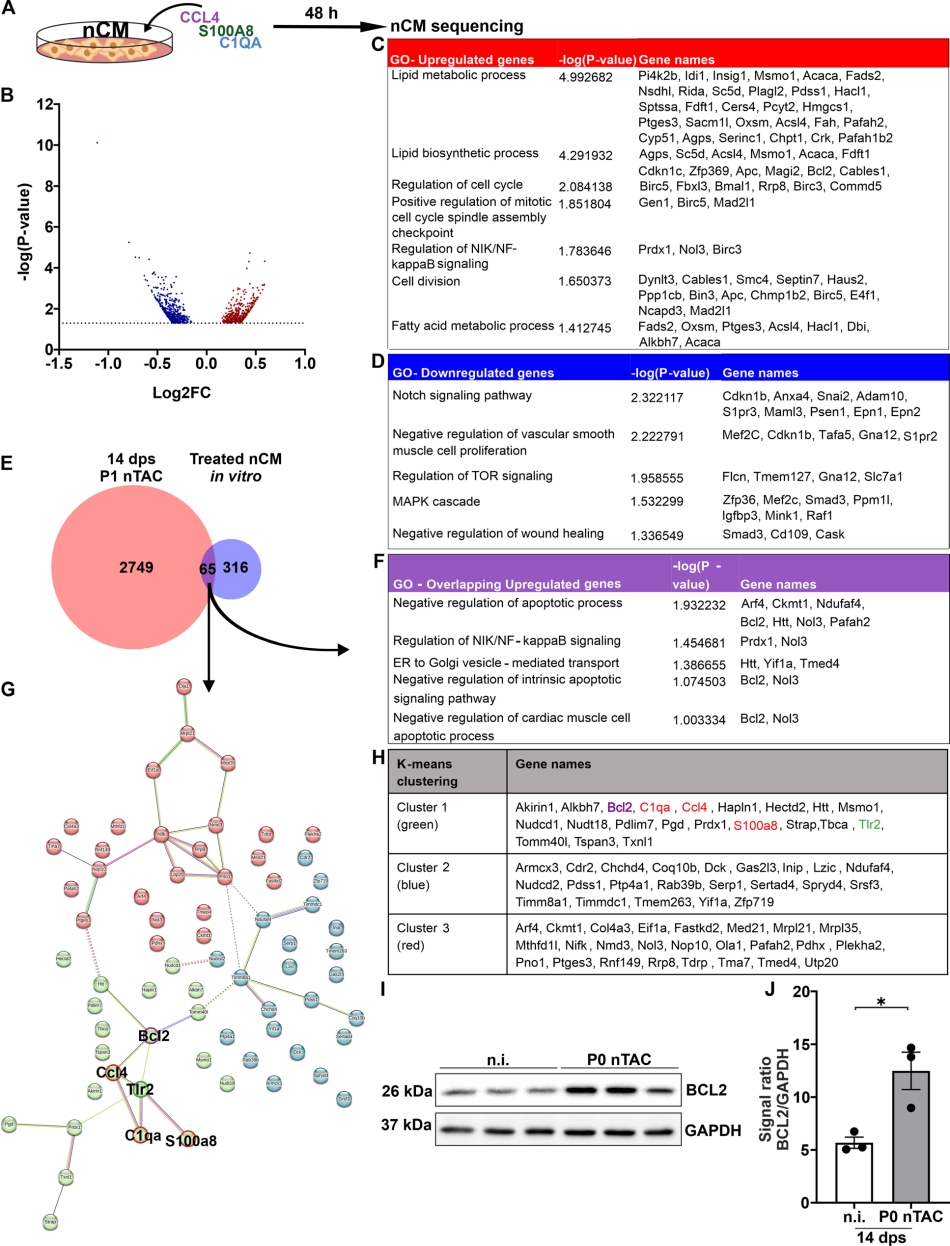
TLR2 activation as a central signaling hub mediating pro-survival and cell cycle re-entry in nCM in vitro and in vivo. **A** Schematic images of the experiment. **B** Volcano plot of regulated genes in nCM upon treatment with Pool3. *n* = 5 per condition. **C-D** Selected GO analysis terms, their corresponding genes, and p-value of upregulated (C) and downregulated (D) genes upon treatment of nCM with Pool3. **E** Venn diagram showing the overlap of upregulated genes at 14 dps P1 nTAC compared to Pool3-treated nCM. **F** Selected GO terms, their p-value, and gene names related to the overlapping genes in 14 dps P1 nTAC and Pool3-treated nCM. **G-H** STRING analysis showing protein-protein interaction and k-means clustering of overlapping genes in 14 dps P1 nTAC and Pool3-treated nCM with gene names in each cluster (H). **I-J** Western blot and quantification of BCL2 in the LV of P14 n.i. and 14 dps P0 nTAC hearts. (*p < 0.05, unpaired t*-*test)

Given the observed effects of Pool3 on enhancing nCM cell cycle re-entry and survival, we aimed to gain insight into the underlying mechanism. Therefore, we conducted a transcriptome analysis of treated nCM using bulk RNA sequencing (Fig. 5A). GO analysis showed that the upregulated genes were primarily related to biological processes such as lipid metabolism, cell cycle regulation, and cell division (Fig. 5B-C). In contrast, the downregulated genes were associated with pathways such as Notch signaling, TOR signaling regulation, MAPK cascade, and negative regulation of wound healing (Fig. 5D). Notably, Cdkn1B (p27), a known inhibitor of nCM proliferation, was among the downregulated genes in the Notch signaling pathway.

To identify key downstream signaling pathways through which Pool3 regulates the identified genes in nCM, we performed STRING analysis to assess protein-protein interactions among the overexpressed genes along with the three proteins (Supplementary Fig. 6A). K-means clustering identified three distinct clusters among the upregulated genes (Supplementary Fig. 6A-B). Cluster 1, which contained the genes encoding the three proteins, showed their direct interaction with TLR2 (Supplementary Fig. 6A). The genes in this cluster are primarily associated with anti-apoptotic genes such as Bcl2, Cxcl10, Nol3 (an apoptosis repressor), and Birc3, indicating that these proteins regulate anti-apoptotic signaling pathways directly through TLR2 (Supplementary Fig. 6B). Clusters 2 and 3 appeared to be indirectly activated. The genes in cluster 2 were related to cell cycle activity and cell division, such as Apc, Cables1, and Birc5, while cluster 3 included genes involved in lipid and fatty acid metabolic processes, suggesting regulation of metabolic pathways as well (Supplementary Fig. 6A-B). Thus, our bioinformatic analysis further underscored the activation of anti-apoptotic and cell cycle re-entry-associated genes in nCM upon treatment with Pool3. Next, we investigated whether similar signaling pathways are activated in vivo upon upregulation of these factors.

### Comparison of upregulated genes in vitro and in vivo after neonatal cardiac injury reveals activation of similar pro-survival and cell cycle re-entry signaling pathways

Next, we aimed to investigate whether Pool3 treatment-induced genes and signaling pathways in nCM are also activated in the heart after P1 nTAC^5^. To this end, we used a Venn diagram to compare the upregulated genes at 14dps P1 nTAC with those in our Pool3-treated nCM (Fig. 5E). Interestingly, we identified 65 common upregulated genes (Fig. 5E), and gene ontology analysis revealed that these are mainly associated with processes such as negative regulation of apoptosis (Fig. 5F).

To investigate the signaling pathway, we performed STRING analysis and studied the protein-protein interaction of overlapping genes along with the three proteins, followed by K-means clustering. This analysis identified three clusters among the upregulated genes both in vivo and in vitro (Fig. 5G-H). The first cluster contained the Pool3 genes, thus appearing to be the initially activated signaling pathway, showing their direct interaction with Tlr2. Genes of this cluster were related to anti-apoptotic genes such as Bcl2 (Fig. 5G-H). BCL2 overexpression in vivo was confirmed by Western blot analysis of the LV from 14dps P0 nTAC and control. (Fig. 5I-J). Immunostaining of control, TLR2 agonist (zymosan), and Pool3-treated nCM also showed high BCL2 staining intensity in vitro (Supplementary Fig. 6C). Additionally, using our recently published single-nucleus RNA-sequencing (snRNA-seq) dataset of nCM from 14dps P0 nTAC and control hearts [27], we confirmed upregulation of Bcl2 among other anti-apoptotic genes in nTAC-Tlr2-positive nCM in the previously reported regenerative/cycling nCM subpopulation (CM2) [27] (Supplementary Fig. 7A).

The second cluster was associated with pro-proliferative genes such as Ptp4a1, which stimulates the G1 to S phase progression during mitosis; Inip, which promotes DNA repair and the G2/M checkpoint, and Ndufaf4, which enhances both cell proliferation and survival (Fig. 5G-H). The third cluster included genes related to the negative regulation of apoptosis, such as Nol3, Arf4, Ckmt1, and Pafah2, as well as mitochondrial translation. Thus, these analyses suggested that endogenous overexpression and secretion of CCL4, C1QA, and S100A8 in the heart upon P1 nTAC activate protective and cell cycle re-entry-associated signaling pathways in nCM, and that it is mediated via TLR2.

Since TLR2 is not a direct receptor for any of the proteins included in Pool3, we performed NicheNet analysis to identify potential indirect signaling pathways through which CCL4, C1QA, and S100A8 may activate TLR2-associated signaling. This analysis suggested indirect links between these ligands and Tlr2 via the following predicted regulatory axes: CCL4–Gnb2–Psmd2–Tlr2, C1QA–Pcbp2–Arhgef7–Tlr2, and S100A8–Trim25–Trp53–Tlr2.

Next, to identify Tlr2-dependent signaling pathways potentially regulating nCM cell cycle re-entry, we re-analyzed our snRNA-seq dataset of nCM from P0 nTAC and control hearts [27]. Given our previous identification of CM2 as a regenerative/cycling CM subpopulation enriched after nTAC, we focused on CM2 cells and compared Tlr2-positive and Tlr2-negative CM2 cells within the P0 nTAC group.

Differential expression analysis of nTAC_CM2_Tlr2_positive versus nTAC_CM2_Tlr2_negative cells identified enrichment of several genes associated with DNA replication, mitotic progression, and cell-cycle regulation. Among the most relevant genes were Cdc6, Ccnf, Rmi2, and Birc5/Survivin (Supplementary Fig. 7B). Cdc6 is involved in DNA replication licensing, Ccnf in cell-cycle progression, Rmi2 in DNA replication/repair-associated processes, and Birc5 in mitotic survival and chromosome segregation. Birc5 was also significantly upregulated in Pool3-treated nCM (Fig. 5B-C). These findings suggest that Tlr2-positive CM2 cells acquire a cell-cycle-permissive and mitotic-survival-associated transcriptional program.

To further investigate potential downstream signaling routes linking Tlr2 to these proliferative genes, we used the NicheNet intracellular signaling and gene-regulatory network as a prior model. Network-based path analysis revealed predicted Tlr2-linked routes to multiple cell cycle re-entry-associated targets. The strongest predicted pathways included Tlr2–Trp53–Cdc6, Tlr2–Trp53–Zfy2–Rmi2, and Tlr2–Trp53–Trim25–Ccnf. When extending the analysis to additional cell-cycle and mitotic regulators, we identified further Tlr2-connected pathways involving Esr1, Lrrk2, Psmd2, and Trp53 as intermediate signaling nodes. These included Tlr2–Esr1–E4f1, Tlr2–Lrrk2–Ncapd3, Tlr2–Lrrk2–Mad2l1, Tlr2–Trp53–Gen1, and Tlr2–Lrrk2–Ppp1r3a–Birc5. However, NicheNet also revealed direct regulation of Bcl2 via Tlr2.

Together, these analyses confirmed our STRING analysis, suggesting that Tlr2-associated signaling is directly linked to prosurvival (e.g., Bcl2) and indirectly to a cell cycle re-entry network involving DNA replication, chromosome organization, spindle checkpoint regulation, DNA repair, and mitotic activity (e.g., Birc5).

To determine whether the proteins activate the same signaling in the heart following MI, we compared the overexpressed genes in the P1 MI heart with those in the treated nCM. This comparison also showed overlapping genes (Supplementary Fig. 7C-E). Further investigation of overlapping genes and protein-protein interaction analysis using STRING yielded the same results, namely activation of an initial pro-survival signaling pathway through Tlr2, including Bcl2, Cxcl10, Birc3, and Apc upon overexpression of the proteins (Cluster 1) (Supplementary Fig. 7D-E). Additionally, we detected two other clusters: Cluster 2 included genes related to lipid biosynthesis, such as Acsl4 and Agps, and cluster 3 included genes related to translation (Eif1a, Eif2, Rpl13) and protein folding (Supplementary Fig. 7D-E).

These findings suggest that Pool3 exerts its beneficial effect on nCM through TLR2, thereby promoting pro-survival and cell cycle re-entry signaling pathways in both pressure overload and MI conditions.

### TLR2 mediates pro-survival and cell cycle re-entry of Pool3 in nCM

**Fig. 6:**
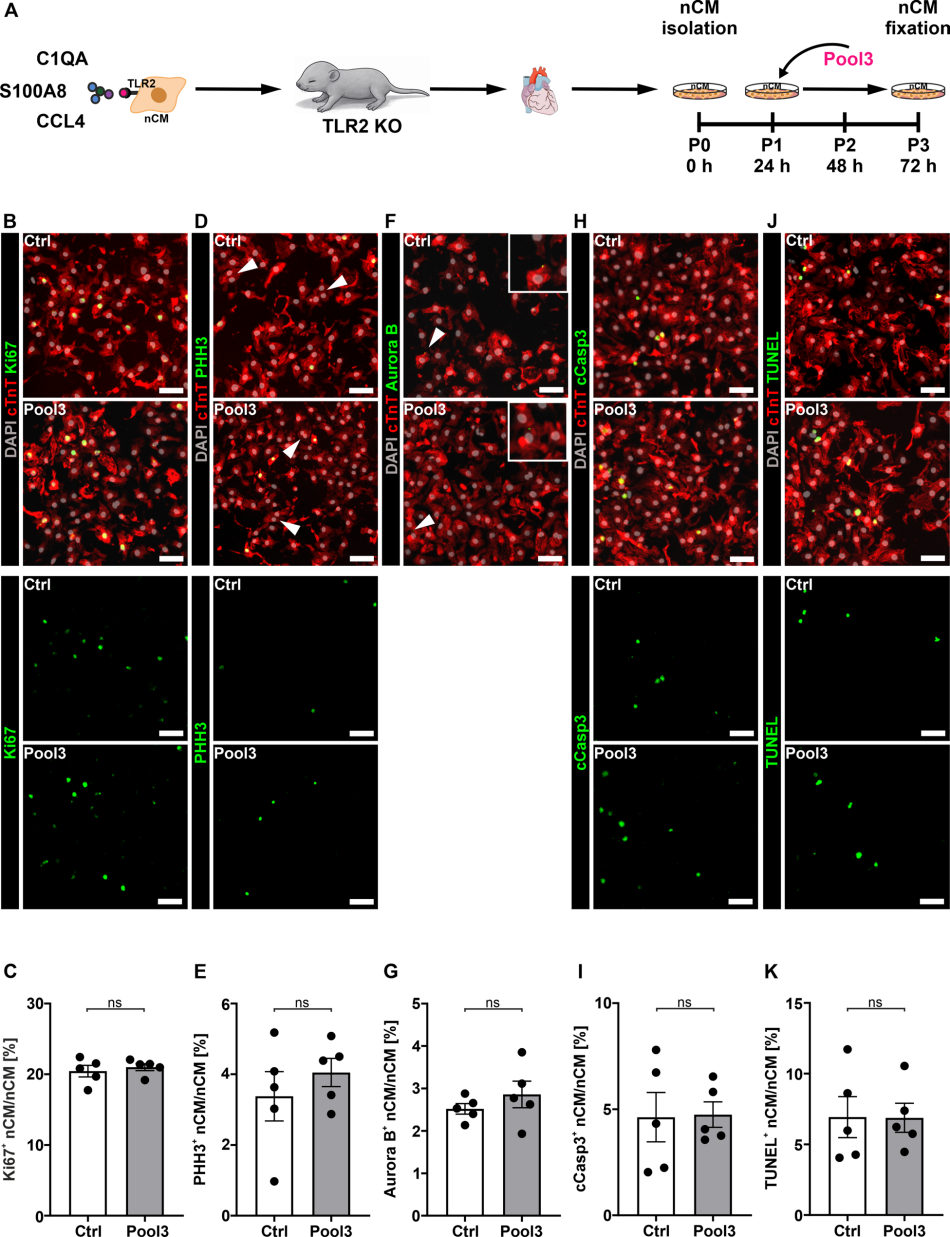
Pool3 does not affect cell cycle activity and apoptosis rate of TLR2 KO nCM in vitro. **A** Schematic image showing the experiment. **B-D** Immunostaining images, single channel, and quantification of Ki67 (**B**-**C**), PHH3 (**D**-**E**), and Aurora B (**F**-**G**) of TLR2 KO nCM in Ctrl and Pool3-treated nCM. **E-F** Immunostaining images, single channel and quantification of cCasp3^+^ (**H**-**I**), and TUNEL^+^ (**J**-**K**) TLR2 KO nCM. Scale bar = 50 μm (ns: not significant; unpaired t*-*test)

To explore the critical role of TLR2 in activating the downstream pro-survival and cell cycle re-entry signaling pathways in nCM upon Pool3 treatment, we employed two strategies, namely the use of TLR2 KO mice/nCM and a TLR2 inhibitor in wild-type nCM. First, we isolated and plated nCM at P0 from TLR2 KO mice, and treated the cells with Pool3 or only medium as a control (Fig. 6A). Interestingly, in full accordance with our bioinformatic analysis, immunostaining and quantification of Ki67, PHH3, and Aurora B in Pool3-treated TLR2 KO nCM did not show any effect on these cells compared to the control (Fig. 6B-G). Likewise, Pool3 treatment of TLR2 KO nCM did not affect the apoptosis rate, as proven by quantitation of cCasp3 immunostaining and TUNEL assay (Fig. 6H-K). To determine whether baseline differences in cell cycle activity or apoptosis contribute to the observed phenotype in TLR2 KO nCM, we compared control and Pool3-treated nCM from TLR2 KO and wild-type CD1 mice. Importantly, baseline cell cycle markers (Ki67, PHH3, and Aurora B) were similar between TLR2 KO and wild-type CD1 control nCM, indicating no major differences in these cell cycle processes under resting conditions (Supplementary Fig. 8A-C). However, after Pool3 stimulation, a significant increase in all three cell cycle markers was seen only in CD1 nCM, whereas TLR2 KO nCM remained unresponsive (Supplementary Fig. 8A–C). In contrast, analysis of apoptosis showed that wild-type CD1 control nCM had higher baseline levels of apoptosis (cCasp3, TUNEL) in vitro than TLR2 KO nCM, which were reduced by Pool3 treatment. This may indicate compensatory pro-survival mechanisms in TLR2-deficient nCM that limit baseline apoptosis (Supplementary Fig. 8D–E). Overall, these findings suggest that the lack of response to Pool3 treatment in TLR2 KO nCM is unlikely due to differences in baseline cell cycle activity and instead highlights a requirement for TLR2 signaling in mediating the observed effects.

**Fig. 7:**
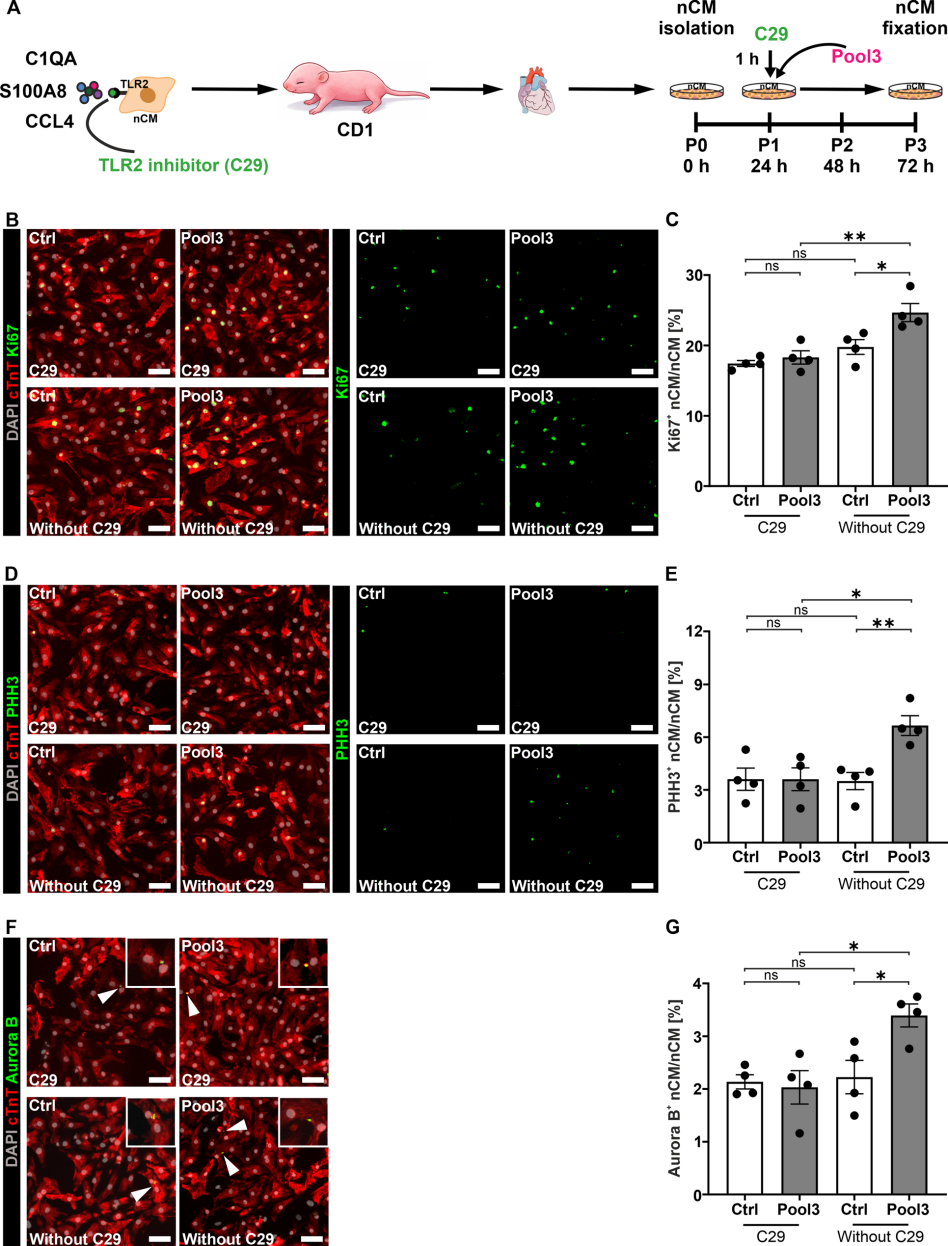
Blocking of TLR2 abolishes Pool3-induced nCM cell cycle re-entry in vitro. **A** Schematic images of the experiment showing the presence of TLR2 in wild-type nCM and inhibition of TLR2 using a specific inhibitor (C29). **B-G** Immunostaining images and quantification of Ki67^+^ (B-C), PHH3^+^ (D-E), and Aurora B^+^ (F-G) nCM. Scale bar = 50 μm, (*p < 0.05; **p < 0.01; ns: not significant, one-way ANOVA with Šídák’s multiple comparisons test)

However, given strain differences between TLR2 KO mice (C57Bl/6) and the wild-type mice (CD1), used in other experiments, together with the established role of TLR2 in cardiac regeneration, we sought to further validate our findings using the TLR2 inhibitor (C29) in wild-type nCM. To this end, nCM were isolated from wild-type CD1 mice at P0, and Pool3 was applied in the presence or absence of C29 (100 µM) (Fig. 7A). We found that C29 blocked the Pool3-induced enhancement of nCM cell cycle re-entry, whereas the positive effects persisted in the control (Fig. 7B-G).

**Fig. 8:**
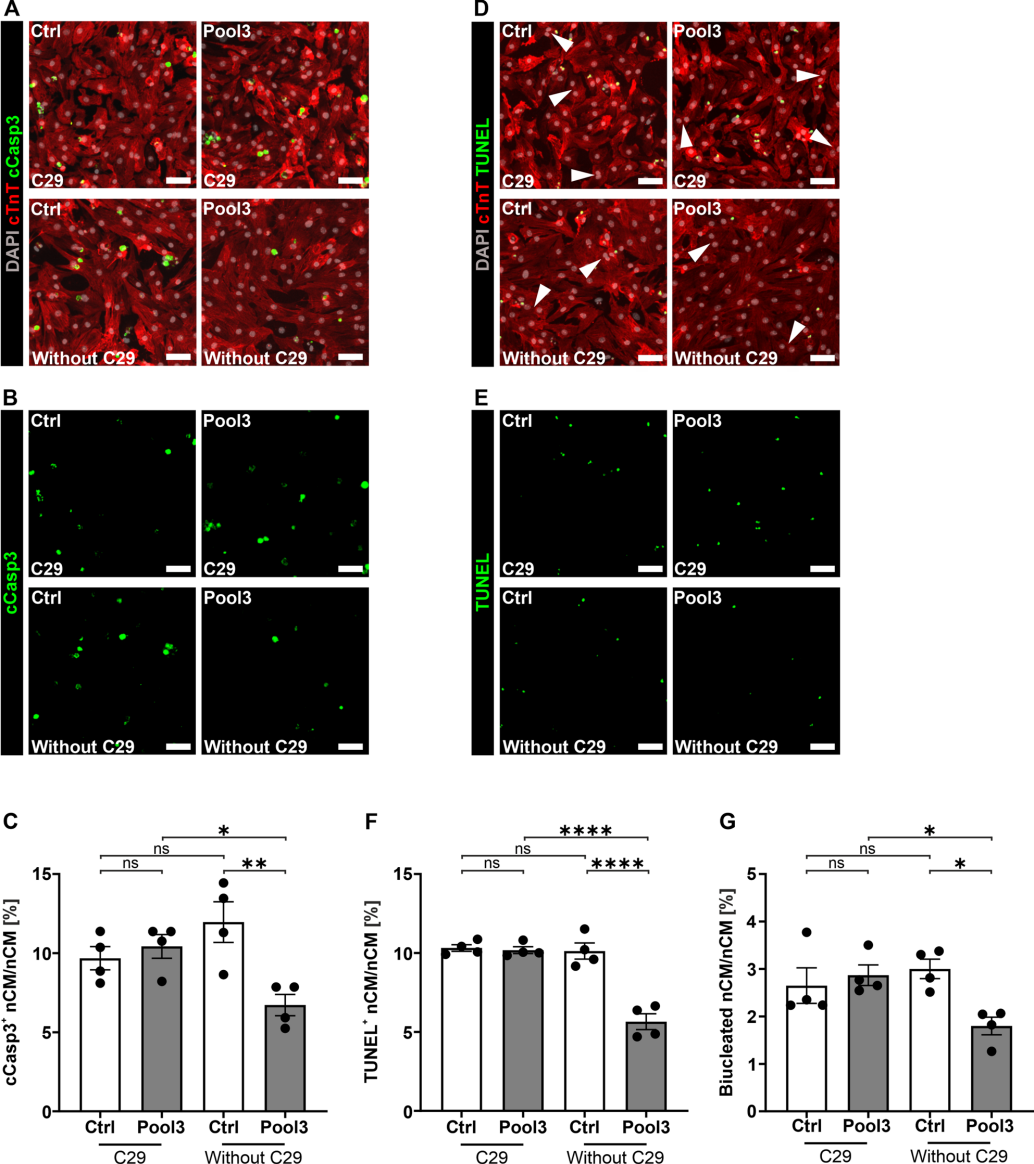
Blocking of TLR2 abolishes the pro-survival effect of Pool3. **A-F** Immunostaining images and quantification of cCasp3^+^ and TUNEL^+^ nCM in Ctrl and Pool3-treated nCM in the presence or absence of TLR2 inhibitor (C29). **G** Quantification of the binucleation rate of nCM in Pool3 and Ctrl-treated nCM in the presence or absence of TLR2 inhibitor (C29). Scale bar = 50 μm, (*p < 0.05; **p < 0.01; ****p < 0.0001; ns: not significant, one-way ANOVA with Šídák’s multiple comparisons test)

Similarly, the observed anti-apoptotic effect was abolished when applying Pool3 in the presence of C29, as there was no difference in cCasp3^+^ and TUNEL^+^ nCM, whereas the apoptosis rates under control conditions were significantly lower upon Pool3 treatment (Fig. 8A-F). We also quantified the binucleation rate to distinguish true cell cycle re-entry from binucleation. Pool3 treatment reduced the binucleation rate compared to the control, indicating an enhanced cell cycle-permissive phenotype, whereas this effect was prevented upon application of the TLR2 inhibitor (Fig. 8G).

As TLR2 signaling often occurs via heterodimerization with TLR1 or TLR6, we assessed the expression of all three Tlrs in nCM at 14dps P0 nTAC and controls using our recently published snRNA-seq dataset [27]. Notably, Tlr2 was the most abundant Tlr across nCM and was markedly upregulated following P0 nTAC (Supplementary Fig. 8F). Interestingly, analysis of Tlr2 expression in nCM using an additional online database [21] revealed that Tlr2 is also upregulated after P1 MI in nCM, but downregulated in adult CM with no change following MI (Supplementary Fig. 8G). High expression of TLR2 in the LV of P0 nTAC hearts compared to controls at 14 dps was confirmed by Western blot analysis (Supplementary Fig. 8H-I). In addition, immunostaining demonstrated higher nCM-specific TLR2 expression in 14 dps P0 nTAC compared to control hearts (Supplementary Fig. 9A). Notably, negligible TLR2 expression in adult CM, compared to nCM, was further confirmed by immunostaining of heart sections (Supplementary Fig. 9A). This pattern further supports a role for TLR2 in maintaining the regenerative capacity of the neonatal heart and highlights its decline with age, concomitant with the loss of regeneration.

Together, these data indicate that Pool3, through activation of TLR2, triggers downstream pro-survival and cell cycle re-entry in nCM. Additionally, the higher expression of TLR2 in nCM compared with adults may facilitate this immune-CM crosstalk, enhancing nCM’s ability to sense and respond positively to inflammatory cytokines such as CCL4, S100A8, and C1QA.

### TLR2 agonist (zymosan) enhances nCM cell cycle re-entry and survival in vitro

**Fig. 9:**
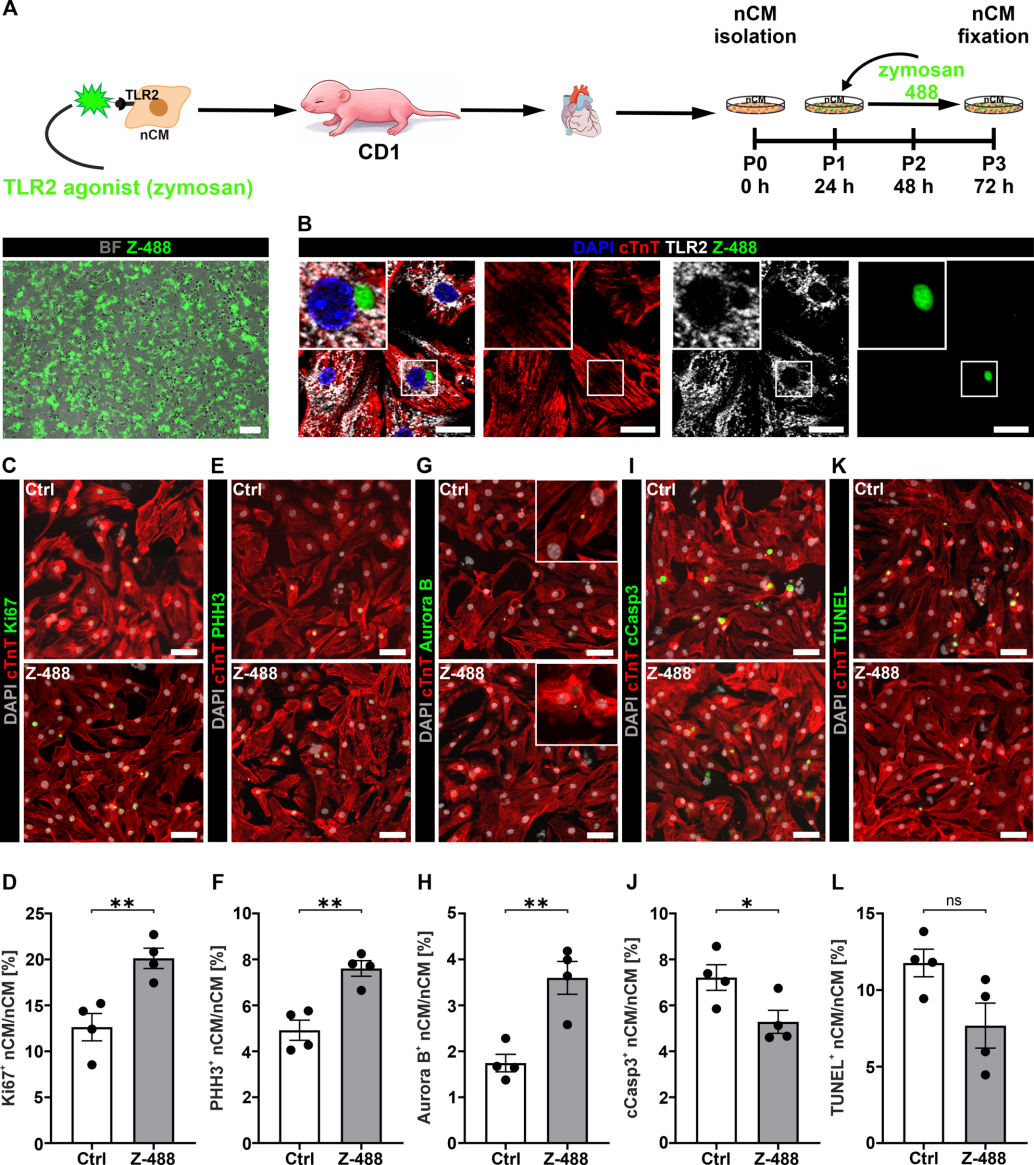
Activation of TLR2 by its agonist (zymosan) enhances the survival and cell cycle re-entry of nCM in vitro. **A** Schematic image of the experiment and an image of unstained nCM treated with zymosan conjugated with Alexa Fluor 488 (Z-488). **B** Immunofluorescence images of nCM stained for cTnT and TLR2 and Z-488 showing the interaction of zymosan with TLR2 on nCM. Insert showing the enlarged image of the zymozan interaction with nCM via TLR2. **C-L** Immunostaining images of Ctrl and Z-488-treated nCM and quantification of Ki67^+^ (C-D), PHH3^+^ (E-F), Aurora B^+^ (G-H), cCasp3^+^ (I-J), and TUNEL^+^ (K-L) nCM. Scale bar in B = 20 μm and C-K = 50 μm (*p < 0.05; **p < 0.01, unpaired t*-*test)

**Fig. 10:**
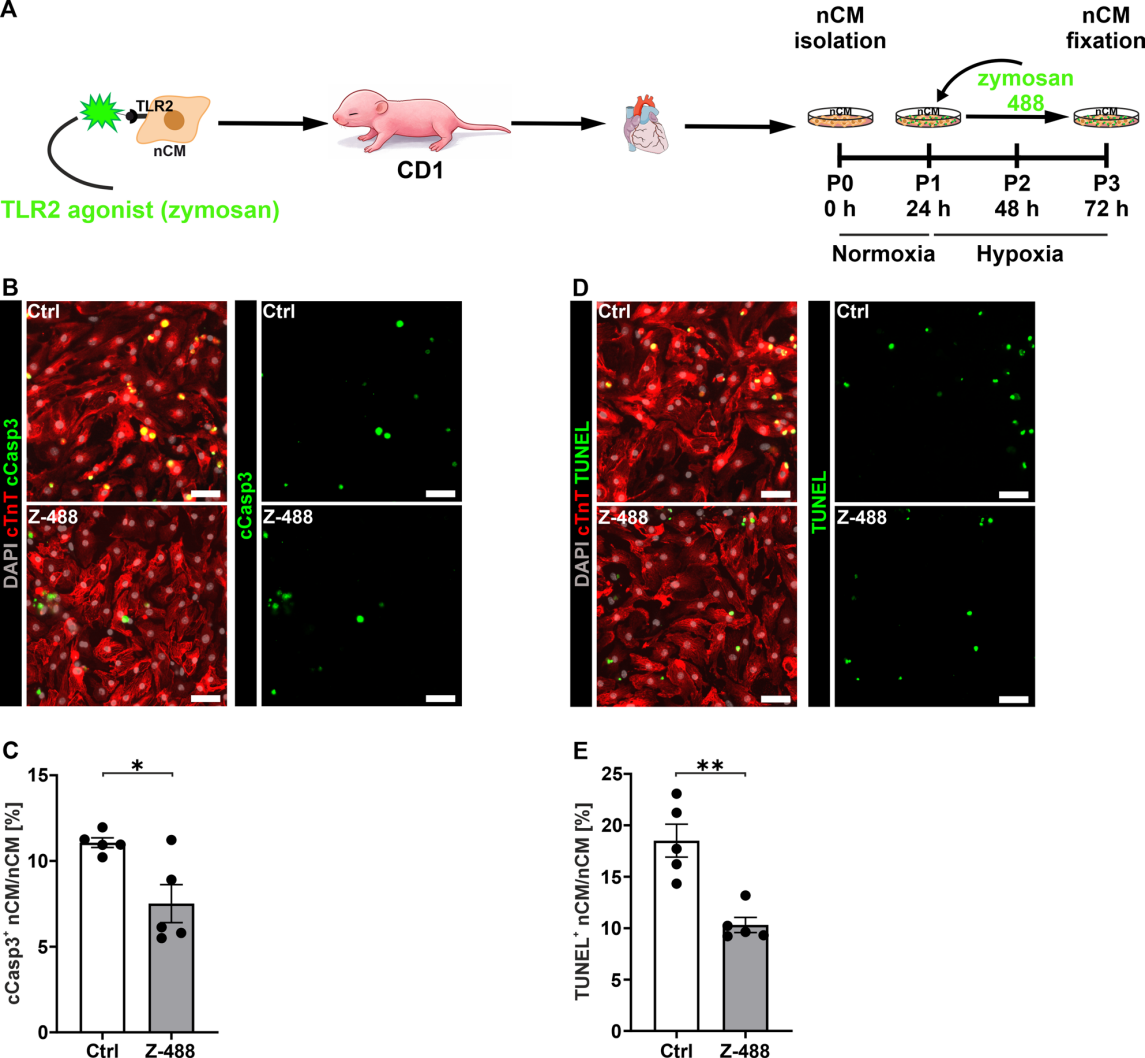
Zymosan reduces the nCM apoptosis rate under hypoxia conditions in vitro. **A** Schematic representation of the experiment culturing and timeline of nCM under hypoxia and treatment with Z-488. **B-E** Immunostaining images and quantification of cCasp3^+^ nCM (B-C) and TUNEL^+^ nCM (D-E) in Ctrl and Z-488-treated nCM. Scale bar = 50 μm (*p < 0.05; **p < 0.01, unpaired t*-*test)

To investigate whether TLR2 activation on nCM alone is the main driver of enhanced nCM cell cycle re-entry and survival or whether the effect is ligand-dependent, we used the TLR2 agonist, zymosan (20 µg/ml) conjugated to Alexa Fluor 488, instead of Pool3 to treat nCM (Fig. 9A). By immunostaining for cardiac troponin T (cTnT) and TLR2, we confirmed that zymosan interacts with TLR2 in nCM (Fig. 9B). Immunostaining for Ki67, PHH3, and Aurora B confirmed the ability of zymosan to enhance cell cycle activity (1.6 fold), mitosis (1.6 fold), and cytokinesis rates (2 fold) (Fig. 9C-H, Supplementary Fig. 9B). Additionally, quantification of the early and late apoptosis rate of nCM using cCasp3 immunostaining and TUNEL assay, respectively, revealed that activation of TLR2 on nCM via zymosan reduced the early, but not significantly the late apoptosis rate (1.4 fold) (Fig. 9I-L, Supplementary Fig. 9C). To confirm the protective effect of zymosan in vitro, which more closely resembles in vivo conditions, we next cultured nCM under hypoxic conditions in the presence of zymosan (Fig. 10A). Quantification of cCasp3^+^ and TUNEL^+^ nCM confirmed that zymosan reduces the nCM apoptosis rate under hypoxia (Fig. 10B-E). Together, these data showed that activation of TLR2 on nCM can promote pro-survival and cell cycle re-entry of nCM as a pro-regenerative mechanism.

### TLR2 deficiency impairs the adaptive response of the neonatal heart to pressure overload

**Fig. 11:**
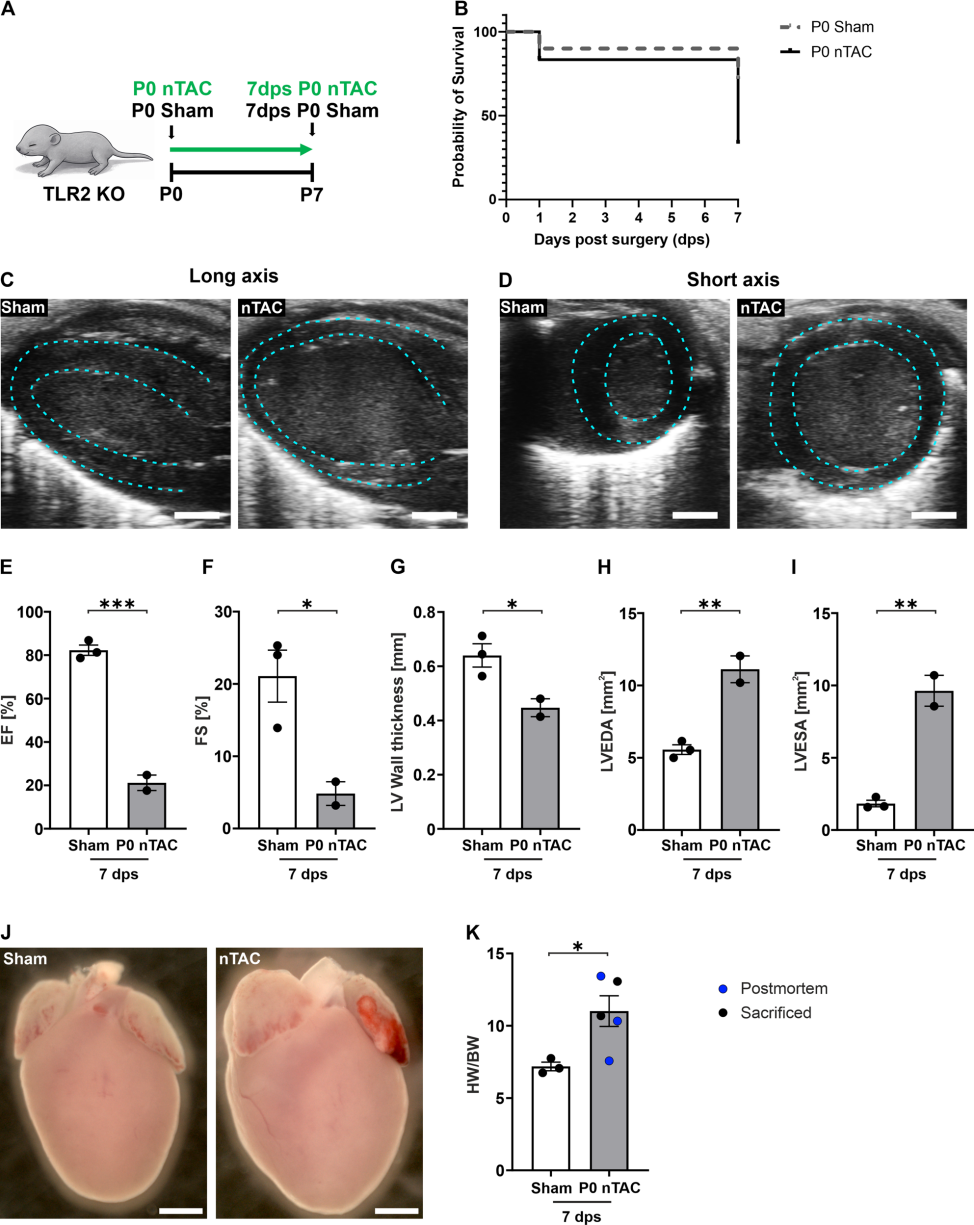
TLR2 KO mice are unable to adapt to pressure overload. **A** Schematic image of the experimental timeline using TLR2 KO mice. **B** Kaplan-Meier survival curve of TLR2 KO sham and P0 nTAC. **C-D** Echocardiographic images showing long (C) and short (D) axis views of the heart in sham and nTAC TLR2 KO mice at 7 dps. **E-I** Quantification of ejection fraction (EF) (E), fractional shortening (FS) (F), LV wall thickness (G), LV end diastolic area (LVEDA) (H), and LV end systolic area (LVESA) (I) of sham and nTAC TLR2 KO mice at 7 dps. **J** Macroscopic images of the hearts in TLR2 KO sham and P0 nTAC at 7 dps. **K** Heart weight/body weight (HW/BW) in sham and P0 nTAC TLR2 KO mice at 7 dps. Scale bar in C-D and J = 1 mm (*p < 0.05; **p < 0.01, ***p < 0.001, unpaired t*-*test). Blue dots indicate tissues collected postmortem, and black dots are those sacrificed

To investigate the role of TLR2 in the adaptive response of neonatal mice to pressure overload, we performed nTAC or sham surgery in TLR2 KO mice at P0 (Fig. 11A). Mortality rate, including cannibalization, was much higher in the P0 nTAC (5/7 = 71%) group compared to the sham group (2/5 = 40%). Unlike the sham, most P0 nTAC mice died by 7 dps (Fig. 11B). Echocardiographic analysis of the surviving mice revealed a marked reduction in ejection fraction (EF) and fractional shortening (FS), aligning with decreased LV wall thickness and increased LV end-diastolic area (LVEDA) and end-systolic area (LVESA) as early as 7 dps (Fig. 11C–I). These findings, which do not align with our previous observations [5] and expectations in wild-type CD1 mice, indicate pronounced maladaptive remodeling in TLR2 KO neonatal mice in response to pressure overload as early as 7 dps. Unlike wild-type CD1 mice, TLR2 KO mice exhibited early cardiac dilation, impaired contractile function, and adverse remodeling. Therefore, we sacrificed the remaining mice at 7 dps and collected data for further analysis from both surviving mice (black dots) and, whenever possible, from freshly deceased/postmortem mice (blue dots) that had not been cannibalized. Heart weight to body weight ratio (HW/BW) was significantly increased in TLR2 KO mice at 7 dps following nTAC (Fig. 11J-K).

**Fig. 12:**
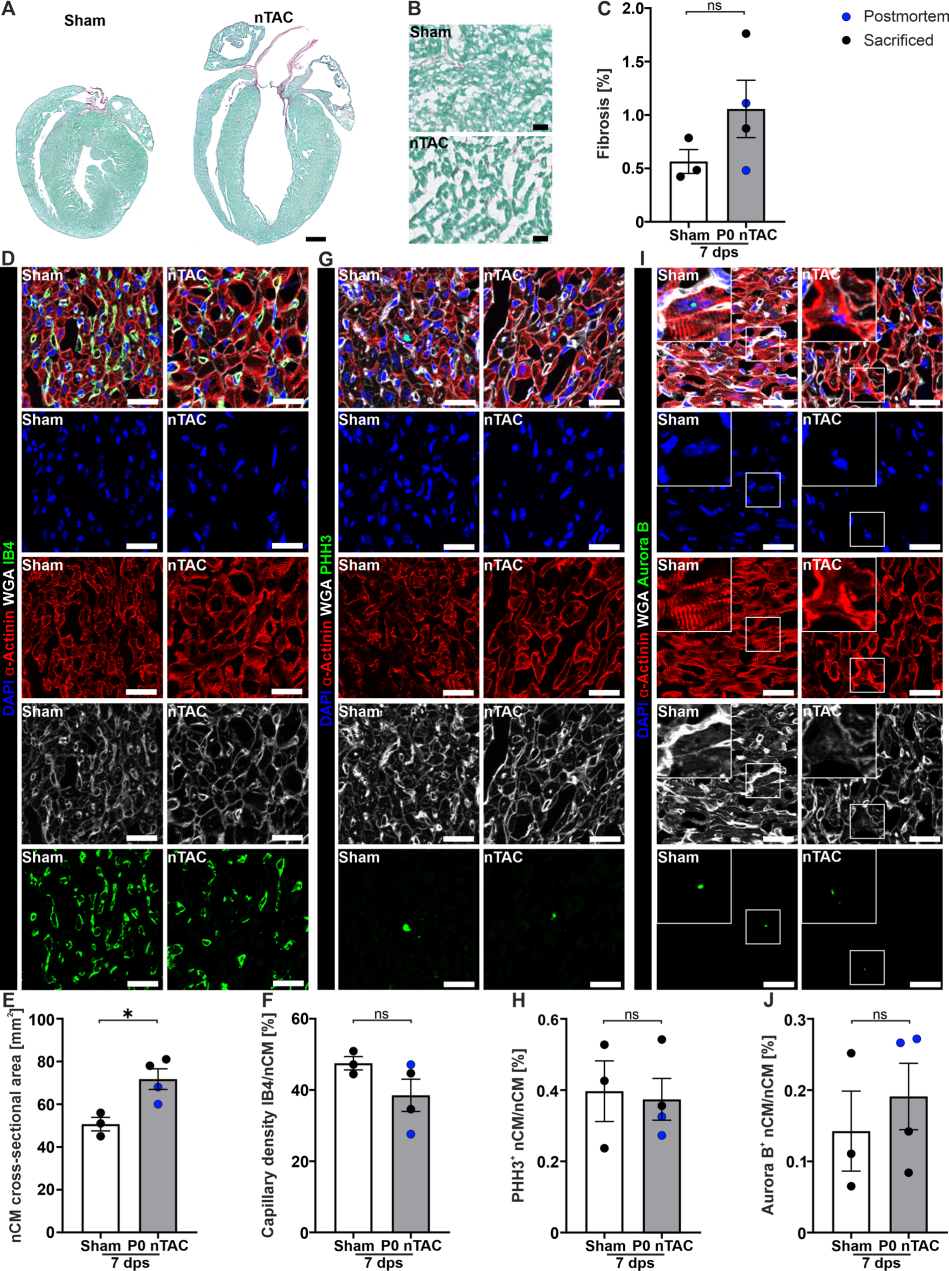
Pressure overload at P0 in TLR2 KO mice induces nCM hypertrophy without enhancing angiogenesis or nCM cell cycle re-entry. **A-C** Representative overview (A) and zoom-in images (B) of Sirius-red/Fast-green–stained heart sections from sham and P0 nTAC TLR2 KO mice and quantification of fibrotic area in the LV (C). **D** Immunostaining images of heart sections from sham and P0 nTAC TLR2 KO hearts for Isolectin B4 (IB4), WGA, DAPI, and α-Actinin. **E-F** Quantification of nCM cross-sectional area (E) and capillary density (IB4/nCM) (F). **G-H** Immunostaining images of heart sections for PHH3, DAPI, and α-Actinin (G) and quantification of PHH3^+^ nCM in heart sections of sham and nTAC TLR2 KO mice at 7 dps (H). **I-J** Immunostaining images of heart sections for Aurora B, DAPI, and α-Actinin (I) and quantification of Aurora B^+^ nCM in heart sections of sham and nTAC TLR2 KO mice at 7 dps (J). Scale bar in A = 500 μm; B, D, G, and I = 20 μm (**p < 0.01; ns: not significant, unpaired t*-*test). Blue dots indicate tissues collected postmortem, and black dots are those sacrificed

Histological analysis of heart sections using Sirius-red/Fast-green staining revealed no significant differences in the extent of interstitial fibrosis at 7 dps (Fig. 12A–C). In contrast, assessment of nCM cross-sectional area demonstrated significant nCM hypertrophy in TLR2 KO hearts upon P0 nTAC (Fig. 12D-E), occurring in the absence of increased capillary density (Fig. 12D, F), which is typically observed at this stage following nTAC in wild-type CD1 mice [5]. Furthermore, quantification of nCM mitosis and cytokinesis rates by immunostaining for PHH3 and Aurora B, respectively, showed that TLR2 KO mice, unlike wild-type CD1 mice, were unable to enhance nCM proliferation/cell cycle re-entry in response to pressure overload (Fig. 12G-J).

Taken together, our data highlight the critical role of TLR2 in mediating the adaptive response of the neonatal heart to pressure overload by promoting nCM cell cycle re-entry and angiogenesis. However, these effects may not be exclusively nCM-autonomous but may also depend heavily on non-nCM-mediated mechanisms, including modulation of the immune response and angiogenic remodeling.

## Discussion

In this study, we aimed to identify novel cardioprotective and regenerative mechanisms that provide long-term benefits and also extend the heart’s regenerative capacity beyond the first week of life. For this purpose, we have chosen a bioinformatic strategy comparing the P1 heart’s immediate response to MI with its response to 2 weeks of pressure overload induced by nTAC, as our earlier data revealed that the nTAC-induced regenerative response persists for at least 2 weeks [5, 27]. We identified three commonly upregulated genes encoding secreted factors, namely S100a8, C1qa, and Ccl4, and found their receptors to be expressed on EC and nCM.

We tested these factors alone and in combination as Pool3 and found that the latter induces cell cycle re-entry, and has pro-survival effects on both EC and nCM, consistent with earlier reported effects in HUVEC [10, 16, 22]. Notably, our work extends these findings by investigating their effect on nCM, a previously unexplored aspect.

To identify underlying mechanisms, we compared in vitro and in vivo datasets and found a partial overlap among upregulated genes. This overlap is biologically plausible given the key differences between the models, as in vivo nCM are exposed for 14 days to a complex multicellular and hemodynamic environment, while the in vitro condition reflects a 48 h exposure to Pool3 alone. Importantly, the overlapping genes probably represent a core nCM-intrinsic signaling program that is strongly activated across different contexts. Mechanistically, we demonstrated that Pool3 mediates its pro-regenerative and protective effects through activation of TLR2 as a central hub on nCM, a conclusion supported by our in vivo data. Across both our in vivo and in vitro datasets, we identified Birc5 and Bcl2 as key downstream mediators of cell cycle re-entry and survival associated with TLR2 activation in nCM, respectively. These findings link innate immune signaling to core molecular programs governing nCM survival and cell cycle re-entry.

Importantly, our bioinformatic analysis suggested that Bcl2 is directly regulated downstream of Tlr2, whereas Birc5 is indirectly linked to Tlr2 through the predicted Tlr2–Lrrk2–Ppp1r3a–Birc5 signaling axis. These findings confirmed that TLR2 activation in nCM is associated not only with anti-apoptotic signaling but also with cell cycle re-entry. This is consistent with a previous study showing that Birc5 determines CM number in the heart, as CM-specific Birc5 KO mice exhibit reduced CM number associated with a lower mitotic rate [28]. Accordingly, Birc5 downregulation has been linked to cell cycle arrest [28, 29], whereas Birc5 overexpression has been reported to inhibit doxorubicin-induced apoptosis in nCM, induce DNA synthesis, and promote cell cycle progression [28, 29].

Thus, our finding provides a mechanistic understanding of previous reports showing that in vivo activation of TLR2 by administration of its agonist, zymosan, induces CM cell cycle re-entry in neonatal mice [30] and zebrafish [31]. Likewise, treatment with TLR2 agonists in adult mice following MI or doxorubicin-induced cardiac injury has been shown to improve cardiac function and promote angiogenesis [32, 33]. Remarkably, zymosan administration after adult MI promotes reparative effects comparable to those achieved with stem cell therapy [32]. However, these beneficial in vivo effects of TLR2 activation have generally been attributed to macrophage-mediated mechanisms, given the high expression of TLR2 in these cells and its established role in regulating immune response. The potential direct effects of TLR2 activation on nCM or EC have remained largely unexplored, as they cannot be selectively evaluated in vivo. Our in vitro findings address this gap by providing clear evidence that TLR2 activation directly induces cell cycle activity and pro-survival signaling in nCM, with comparable signaling implications in EC. TLR2 activation in EC is known to exert pro-angiogenic effects by promoting vessel growth, vascular remodeling, endothelial invasion, and adhesion. Previous studies have further implicated TLR2 in EC-mediated angiogenic responses to diverse stimuli, including tumor progression, as well as in cardiac protection during aging and chronic stress [34, 35]. Consistent with these observations, we found that pressure overload induced at P0 (P0 nTAC) in TLR2 KO mice failed to trigger the angiogenic response typicaly observed in wild-type CD1 hearts. Given that angiogenesis is essential for efficient cardiac regeneration and nCM cell cycle re-entry, these findings suggest that Pool3-mediated TLR2 activation may also promote cardiac regeneration in vivo through indirect, non-cell-autonomous mechanisms involving EC. In line with our findings, adult TLR2 KO mice have been shown to develop age-related cardiac dysfunction with increased fibrosis and CM death, and exhibit aggravated hypertrophy and maladaptive remodeling under pressure overload [36]. Similarly, we showed that P0 TLR2 KO mice had higher mortality and were unable to mount an adaptive response to pressure overload via enhanced nCM proliferation/cell cycle re-entry and angiogenesis. Instead, they exhibited maladaptive remodeling as early as 7 dps, characterized by cardiac dilation, impaired cardiac function, and heart failure, accompanied by nCM hypertrophy. However, similar to the in vivo experiments described above, this maladaptive response to nTAC in TLR2 KO mice cannot be explicitly attributed to an nCM-dependent mechanism. Non-nCM-autonomous elements, such as impaired immune responses, macrophage polarization, and angiogenesis, may also play a key role.

Another study also showed that TLR2 blockade in rat nCM induces apoptosis under oxidative stress, reinforcing its anti-apoptotic role [37]. Although TLR2 KO experiments were performed in C57Bl/6 background mice, we independently confirmed the requirement for TLR2 signaling using a blocking antibody approach in wild-type CD1 nCM. The consistency of these results across different genetic backgrounds supports the reliability of the identified TLR2-dependent signaling pathway. Collectively, our findings identify TLR2 as a central signaling hub mediating Pool3-induced pro-survival and cell cycle re-entry of nCM. Importantly, this functional role does not imply that TLR2 is the direct receptor for Pool3 components, nor does it exclude the involvement of other established receptors for individual ligands, such as TLR4. Indeed, TLR4 may exert redundant or complementary functions. Thus, individual blockade of canonical ligands including TLR4 should be addressed in future studies. Accordingly, our bioinformatic analysis confirmed indirect activation of Tlr2 via CCL4–Gnb2–Psmd2–Tlr2, C1QA–Pcbp2–Arhgef7–Tlr2, and S100A8–Trim25–Trp53–Tlr2 axes.

Notably, the absence of parallel induction of Tlr1 or Tlr6 does not prevent functional TLR2 signaling. Baseline expression of these receptors may be sufficient to enable signaling, and TLR2 has been shown to engage different co-receptors depending on the ligand context [38]. Therefore, selective upregulation of Tlr2 may serve as a sensitization mechanism, enhancing responsiveness to Pool3-associated ligands without requiring the simultaneous induction of all binding partners. Overall, our in vitro and in vivo data consistently support a model in which nCM primarily activate a TLR2-centered signaling pathway in response to stress or regenerative signals.

Moreover, FB and immune cells also express Pool3 ligands and pattern-recognition receptors, including TLRs, and therefore likely contribute to signal propagation in vivo. However, this multicellular involvement reflects the physiological regenerative niche rather than a confounding factor. Our in vitro experiments with isolated nCM and EC show cell-intrinsic responsiveness to Pool3, while in vivo, additional paracrine inputs from FB and immune cells are expected to enhance or sustain these signals through non-cell-autonomous mechanisms. Dissecting the relative contributions of individual cardiac cell types will require future cell-type-specific perturbation approaches. We also acknowledge the limitations of the in vitro study and the concentrations used, which were intended to model local paracrine signaling rather than systemic exposure. Future time-resolved quantitative protein measurements in tissue lysates could help define the physiological abundance and temporal regulation of the identified secreted factors after surgery.

Altogether, our findings uncover a previously unrecognized crosstalk axis during cardiac regeneration, in which CCL4, S100A8, and C1QA act in concert to activate TLR2-mediated pro-survival and cell cycle re-entry in nCM. This signaling cascade appears to be essential not only for the initial regenerative response after myocardial injury, but also for adaptive remodeling during ongoing pressure overload. Our data further suggest that TLR2 functions as a key regulator of regenerative signaling in nCM, while this regenerative activity is lost during postnatal maturation and adulthood, potentially contributing to the limited proliferative capacity of adult CM. These insights establish TLR2 as a crucial molecular hub linking inflammatory signals to innate regenerative responses in the neonatal heart. Future studies should determine whether re-expression of TLR2 in adult CM, particularly in the presence of its ligands, is sufficient to restore regenerative competence. Together, these findings open new therapeutic opportunities to re-establish neonatal-like regenerative mechanisms in the adult heart, potentially reducing cardiovascular morbidity and mortality.

## Methods

### Mouse lines

Crl: CD1(ICR) wild-type mice at P0-P1 were used for nCM and EC isolation. The mice had free access to water and food and were kept at a room temperature of 22 °C *±* 2 °C with a 12 h day and night cycle. We also used pups (P0-P1) from a transgenic mouse line αMHC-H2BmCherry x CAG-eGFP-anillin [25, 26] to assess nCM cell cycle re-entry rate, and for the TLR2 KO mice/nCM, we used B6129-*Tlr2*^*tm1Kir/J*^[39]. nTAC surgery and echocardiography were performed as described previously [5, 6]. Animal experiments were performed in accordance with institutional guidelines and approved by the ethics board (81-02.04.2020.A463, Landesamt für Natur, Umwelt und Verbraucherschutz (LANUV), Nordrhein-Westfalen (NRW), Germany).

### Cell isolation for in vitro study

For each nCM biological replicate, 2–3 hearts were pooled. The neonatal hearts at P0-P1 were dissociated using a neonatal heart dissociation kit (Miltenyi, 130-098-373), and nCM were isolated according to the manufacturer’s protocol using the nCM isolation kit (Miltenyi, 130-100-825). Each well of a 96-half-well plate (Greiner bio-one, 675986) was incubated with 50 µl of 0.1% gelatin for 15 min to 2 h at 37 °C before seeding the cells. After nCM isolation, 20,000 cells were seeded in each well. The culture media for nCM contained 2% heat-inactivated FCS, 1% Penicillin/ Streptomycin, and 1% non-essential amino acids (NEAA) in IMDM medium. All cells were cultured in a humidified cell incubator at 37 °C with 5% CO_2_ concentration.

### nCM and EC co-isolation

The neonatal hearts at P0-P1 were dissociated using a neonatal heart dissociation kit (Miltenyi, 130-098-373). For co-isolation of primary nCM and EC, a neonatal endothelial mouse cell isolation kit was used (Miltenyi, 130-106-768), and only the first step of the kit was performed to remove non-EC using the non-cardiac EC depletion cocktail mouse antibody solution (Miltenyi, 130-104-183). The rest of the cells containing nCM and EC were quantified and seeded in a gelatin (0.1%) coated 96-half-well plate.

### Treatment of nCM and EC

24 h after seeding, primary nCM (and EC) were treated with the desired protein for an additional 48 h. Initially, primary nCM was used to screen for the appropriate protein concentration and duration. For this purpose, nCM were treated for either 24–48 h with following protein concentrations C1QA (Cusabio, CSB-EP003637MOa0) with 5, 10, 20, 50, 200 ng/ml, CCL4 (biotech, 451-MB/CF) with 10, 30, 50, 100 and 200 ng/ml and S100A8 (Biotechne, 9877-S8) with 20, 50, 100, 200 and 500 ng/ml. Based on the best results observed on enhancing nCM cytokinesis, the following concentrations were selected for all of the experiments: CCL4 (100 ng/ml), S100A8 (50 ng/ml), and C1QA (200 ng/ml) following 48 h incubation.

The proteins were diluted in serum-free IMDM medium to a concentration twice the final treatment concentration. 50 µl of this solution was then added directly onto the cells without removing the medium (50 µl). This was done to minimize the loss of cells, which would occur when removing the medium. The cells were fixed using 4% PFA after 48 h of treatment. For the TLR2 blocking experiment, after 24 h of nCM seeding, nCM were treated with TLR2 inhibitor (C29) (100 µM in DMSO, MCE, HY-100461) or without as a control for 1 h. Then, nCM were treated with Pool3 for an additional 48 h. For TLR agonist treatment 24 h after seeding, nCM were treated with zymosan-488 (20 µg/ml in PBS with 2 mM sodium acid, Invitrogen, Z-23373) for an additional 48 h. For hypoxia, after 24 h of seeding and cultivation under normoxic conditions, nCM were treated with zymosan and kept under hypoxic conditions (5% O_2_, 5% CO_2_) for an additional 48 h.

### RNA isolation, sequencing, and bioinformatics

After treatment, cells were washed once with PBS and immediately lysed for RNA isolation according to the manual of the RNeasy Plus Micro Kit (Qiagen, 74034). Quantitative measurements were initially performed using the Spark multimode-microplate reader (Tecan, 30086376). Quality measurements were performed using the 2100 Bioanalyzer (Agilent, G2939BA). For quantity assessment, the RNA 6000 Pico Reagents kit (Agilent, 5067 − 1513) as well as the RNA Pico Chip (Agilent, 5067 − 1511) were used. For each condition (control and Pool3), *n* = 5 biological replicates were sequenced.

For bioinformatic analysis, we used the Galaxy platform (Freiburg Galaxy Project) [40]. RNA sequencing reads were mapped using RNA STAR [41], followed by counting reads per gene using FeatureCounts [42]. Differentially expressed genes were identified by DESeq2 [43] with a p-value < 0.05. For data visualization, normalization, cluster analysis, and protein-protein interaction, Heatmapper Expression [44], BioVenn [45], STRING analysis [46, 47], and NicheNet [48] were used. GO analysis was performed by DAVID [49]. GO-terms with a significance of *p* < 0.05 were selected.

### HUVEC culture

HUVEC (Lonza, C2519A) in passages 2–4 were used in this study and cultured using EC proliferation medium (ECGM) (Provitro, 2010001) containing 1% of Penicilin/Streptomycin, including supplements (Provitro, 2180001). HUVEC in ECGM were seeded into a 96-well plate with 5,000 cells per well. After 24 h, the medium was changed to IMDM containing 1% FCS overnight before treatment. The next day, the medium was changed to 2% IMDM, including the proteins. The treatment was stopped after 48 h, and cells were immediately fixed.

### Migration assay using HUVEC

Migration assays were performed in 24-well plastic plates, which were coated with 5% gelatin for 15 min at 37 °C. HUVEC in 300 µl ECGM were seeded with 20,000 cells per well and were cultured until cells were confluent. Confluent cells were scratched with a yellow pipette tip in a straight line across the whole well. After the scratch was made, the cells were washed with PBS, and the treatment medium (proteins diluted in 2% IMDM) was added. Cells were then placed in a live cell imaging chamber and imaged overnight using the Keyence BZ-X800E microscope.

### Angiogenesis assay using HUVEC

The tube formation assay was performed by coating each well of a plastic 24-well cell culture plate (Corning, 353047) with 300 µl of Matrigel. Matrigel was solidified by placing the plate at 37 °C for 15 min. HUVEC in 150 µl of 1% IMDM were seeded with 60,000 cells in each Matrigel-coated well. Proteins were diluted to double the treatment concentration in the same medium, and 150 µl of the diluted protein was added to the cells to reach the desired concentration. Imaging was done after treatment overnight (14 h) using the Zeiss Axio Vert.A1 microscope.

### Immunohistochemistry of heart sections

Whole hearts were fixed with 4% PFA for 1 h (adult: 2 h) at 4 °C after a short incubation in 0.5 M KCl solution (neonatal). After fixation, the hearts were briefly washed in PBS, and then incubated overnight in 30% (adult 20%) sucrose solution at 4 °C. Finally, the hearts were embedded in embedding medium (Tissue-Tek^®^ O.C.T. Compound, Sakura Finetek, 4583). For long-term storage, the hearts were stored at -80 °C. Cryosections with a thickness of 7 μm were prepared and stored at -20 °C. For immunofluorescence staining, the tissue sections were fixed with 4% PFA for 10 min. After washing three times with PBS, the sections were permeabilized with 0.3% Triton-X for 20 min. Subsequently, the sections were washed three times with PBS and blocked with 5% donkey serum for 20 min. Afterwards, the sections were incubated with the primary antibodies (Ki67: 1:200, Invitrogen, 14-5698-82; PHH3: 1:200, Merck, 06-570; Aurora B: 1:200, Merck, A5102; TLR2: 1:200, Biorbyt, orb411837; S1008A: 1:200, Invitrogen, PA5-79948; C1QA: 1:200, Invitrogen, PA5-102927; CCL4: 1:200, R&D Systems, AF-451-NA; α-Actinin: 1:400, Merck, A7811, PDGFRα: 1:200, BD Bioscience, 558774 and R&D systems, AF1062; CD45: 1:200, Cell signaling, 70257 and 1:1000, Merck, 05-1416), diluted in blocking solution, overnight at 4 °C. The next day, the sections were washed three times with PBS and incubated with the secondary antibodies for 1 h. The secondary antibodies conjugated to Alexa Fluor 488, Alexa Fluor 555, or Alexa Fluor 647, respectively (all 1:400, Jackson Immuno Research, West Grove, USA), IB4 (1:100, Biozol, FL-1201), or WGA (1:1,000, Vector Laboratories, FL-1021) were diluted in PBS containing DAPI (Thermo Fisher Scientific, R37606). Finally, the sections were washed three times with PBS and mounted using mounting medium (Polysciences, 18606). The stained sections were then dried overnight at room temperature.

### Sirus-red/Fast-green staining

Tissue sections were first rehydrated in deionized water for 5 min and then fixed in preheated Bouin’s solution at 55 °C for 1 h. Next, the tissue sections were washed with deionized water. All the following steps were performed at room temperature. The tissue sections were stained in a 0.1% Fast Green staining solution for 10 min. Next, the tissue sections were washed three times with deionized water and incubated for 2 min in 1% acetic acid. Afterwards, sections were washed three times with deionized water and were stained with 0.1% Sirius Red staining solution for 30 min. Then, sections were washed with deionized water, followed by dehydration using a series of increasing isopropanol concentrations (70%, 90%, 2 × 100%) for 3 min each. Finally, the tissue sections were incubated three times for 5 min each in xylene and mounted with mounting medium (Merck, 1.07961.1000).

### Western blot

To isolate protein samples from heart tissue, the tissue was first lysed using a homogenizer (Takara Bio Inc., 9791 A). For this purpose, LV tissue was mechanically homogenized with cold RIPA buffer (Thermo Fisher Scientific, 89901, supplemented with proteinase and phosphatase inhibitors) using special mixing rods. Homogenized and lysed tissue was then incubated for 20 min on ice. The samples were then centrifuged at 3600 rpm and 4 °C for 5 min, and the supernatant containing the proteins was collected. Protein determination was performed using the Pierce™ BCA Protein Assay Kit (Thermo Fisher Scientific, 23255). Absorbance was measured at 562 nm using the SPARK^®^ Multimode Microplate Reader and SPARK i-control™ software. For protein expression analyses, the proteins were first separated by size using SDS-PAGE in a 10% separating gel under reducing conditions. A total concentration of 40 µg of protein was loaded. The proteins were separated at 75 V for 30 min in the loading gel and subsequently for 2 h at 100 V in the separation gel using cold running buffer (Bio-Rad, 1610732). Afterwards, proteins were transferred to a 0.45 μm PVDF membrane (Advansta, 541047) at 100 V for 45 min with cold transfer buffer (Advansta Inc., R-03090-D50). The membrane was subsequently blocked with 5% milk powder (in TBS-T) for 1 h at room temperature. Subsequently, the membrane was incubated with primary antibodies (BCL2: 1:1,000, Abcam, ab182858; TLR2: 1:1,000, Abcam, ab209216), diluted in a 5% milk powder block, overnight at 4 °C on a shaker. Next, the membrane was washed three times for 5 min with TBS-T and incubated with the secondary antibodies (rabbit HRP: 1:10,000, Merck, A0545; 555-GAPDH: 1:3,000, Invitrogen, MA5-15738-A555), diluted in a 5% milk powder block, for 1 h at room temperature. For HRP-catalyzed detection, the membrane was incubated with Pierce™ ECL Western Blotting Substrate (Thermo Fisher Scientific, 32106) for 2 min. The protein bands were detected using the ChemiDoc™ MP Imaging System. The analysis was performed using the Image Lab software from Bio-Rad. GAPDH was used for normalization of protein expression.

### Immunohistochemistry of cultivated cells

Cultured cells were washed with PBS once and fixed with 4% PFA for 20 min. Then, cells were washed three times with PBS and permeabilized with a 0.2% Tween-20 solution in PBS for 5 min. Next, cells were washed three times with PBS and were subsequently blocked with 5% donkey serum in PBS for 30 min. The primary antibodies (Ki67: 1:200, Invitrogen, 14-5698-82 or, abcam, ab16667; PHH3: 1:200, Merck, 06-570; Aurora B: 1:200, Merck, A5102; cCasp3 (Asp175): 1:200, Cell Signaling, 9661 L; cTnI: 1:200, abcam, ab56357; cTnT: 1:200, abcam, ab8295; CD31/Pecam-1: 1:200, Biogenex, MU241-UC, TLR2: 1:200, Biorbyt, orb411837; BCL2: 1:200, Invitrogen, MA5-11757) were diluted in 5% donkey serum and applied to the cells overnight at 4 °C. The next day, the cells were washed three times with PBS, and the secondary antibodies were applied for 1 h at room temperature. The secondary antibodies conjugated to Alexa Fluor 488, Alexa Fluor 555, or Alexa Fluor 647, respectively (all 1:400, Jackson Immuno Research, West Grove, USA) or IB4 (1:100, Biozol, FL-1201) were diluted in PBS containing DAPI (Thermo Fisher Scientific, R37606). Finally, the cells were washed with PBS and imaged.

### TUNEL assay

For staining of apoptotic cells, the TUNEL assay (TdT-mediated dUTP-biotin nick end labeling) was used according to the manufacturer’s protocol for the in situ cell death detection kit (Roche 11684795910). nCM were also co-stained with cTnT.

### nCM and EC cell cycle activity/apoptosis assay

For in vitro analyses, each biological replicate consists of a pool of 2–3 hearts of neonatal mice (P0-P1). For each experiment, 4–6 biological replicates were used (described by the n-number), and 2–3 technical replicates for each biological replicate were used for each treatment condition. The resulting numbers of technical replicates of one biological replicate were averaged for evaluation. For in vivo analyses, one heart represents one biological replicate. For quantification, 6–9 images with a 20x objective (in vitro) or 5 images with a 40x objective (in vivo) (Axio Observer from Zeiss, Germany) were quantified. For Aurora B staining, only midbody localization between two nuclei was quantified to ensure assessment of cytokinesis. Although we applied strict criteria and this approach is widely used, we cannot fully exclude the possibility of binucleation or endoreplication. For the Quantification of apoptosis, 6–9 images of the cCasp3 (early apoptosis) and TUNEL assay (late apoptosis) staining were taken with a 20x objective of the Keyence BZ-X800E microscope (Keyence, Japan) or Axio Observer from Zeiss. The quantification was performed manually or using the Keyence or Zeiss analysis software (BZ-X800 Analyzer, Zen 3.1) with manual input to differentiate mono- from binucleated cells. All cells per image were counted. The number of cells varied in vitro, averaging 135 cells per image.

### HUVEC analysis

Images of HUVEC were taken with a 10x objective (Zeiss Axio Observer microscope), and 6 images were evaluated per condition, with approximately 300 cells per image (70,599 cells for Ki67 evaluation and 66,042 cells for cCas3 evaluation). The evaluation was done by using the analysis extension of the Zen 3.1 software. Here, positive signals were automatically detected by the software, and cell numbers were also automatically counted. Manual input was required to delete false-positive detections of background staining.

### Migration assay analysis

The migration assay was evaluated by using time-lapse microscopy and measuring the area of the scratch directly after the initial manipulation (t0) and 15 h afterward (t15). The area was determined with the BZ-X800 Analyzer software (Keyence, Japan). Images were taken every 15 min overnight with a 4x objective by the Keyence BZ-X800E microscope.

### Angiogenesis assay and analysis

For in vitro analyses, Images of each condition were taken with a phase contrast objective (20x). For qualitative and quantitative assessment of the degree of angiogenesis, the total number of closed tubes and branching points was determined in each image and averaged. For in vivo analyses, 5 images of IB4 staining per biological replicate (LV) were taken with a 40x objective (Axio Observer from Zeiss, Germany). For quantification, the number of IB4^+^ cells per nCM was determined.

### In vivo analysis of CCL4^+^, S100A8^+^ and C1QA^+^ cells

Overview images of stained heart sections were taken with a 20x objective (Zeiss Axio Observer microscope). CCL4^+^, S100A8^+^, and C1QA^+^ cells were counted in whole heart sections and were quantified per mm^2^.

### Fibrosis analysis

Overview images of the Sirius-red/Fast-green staining (three heart sections per biological replicate) were taken with the 20x objective of a Keyence BZ-X800E microscope (Keyence, Japan). Afterwards, the red and green areas were measured in the LV using the ImageJ software, and the percentage of red signal to total signal was calculated. Large blood vessels were excluded to only analyze interstitial fibrosis.

### Statistics

Data sets were analyzed using either an unpaired t-test for comparisons between two data groups or one-way ANOVA followed by Šídák’s multiple comparisons test for comparisons among multiple groups. Statistical significance was indicated as follows: * *P* *≤* 0.05; ** *P* *≤* 0.01; *** *P* *≤* 0.001; **** *P* *≤* 0.0001.

## Supplementary Information

Supplementary Material 1: Figure S1. S100A8, C1QA, and CCL4 appear to be among the secreted factors commonly upregulated shortly after P1 MI and 14 dps P0 nTAC but not 3 dps P0 nTAC. A-B) Molecular function and cellular component analysis with the corresponding gene numbers overlapping in 1 dps P1 MI and 14 dps P1 nTAC. C-H) Immunostaining images and quantification of S100A8+, CCL4+, and C1QA+ cells at P3 in n.i. and P0 nTAC hearts. Scale bar = 20µm (*p<0.05, unpaired t-test).

Supplementary Material 2: Figure S2. Ccl4, S100a8, and C1qa are ligands upregulated in P1 MI and nTAC with expressed receptors in nCM and EC. A) Known receptors for CCL4, S100A8, and C1QA. B) STRING analysis showing protein-protein interaction of the proteins and their receptors. C-D) Gene expression of the receptors of CCL4, S100A8, and C1QA in nCM and EC from P1 sham hearts. E-F) Heatmap showing expression of Ccl4, S100a8, and C1qa in FB and immune cells at 2 dps P1 sham and P1 MI using public database21. G-I) Comparison of the expression level of S100a8, C1qa, and Ccl4 in FB compared to immune cells 2 dps P1 MI using the public database21. (****p<0.0001, unpaired t-test).

Supplementary Material 3: Figure S3. Testing various concentrations of the proteins on nCM identified the optimal concentration for the induction of nCM cell cycle re-entry, without affecting the proliferation rate of HUVEC. A-B) Testing the effect of various concentrations of C1QA on nCM mitosis and cytokinesis. C-D) Testing the effect of multiple concentrations of S100A8 on nCM mitosis and cytokinesis. E-F) Testing the effect of various concentrations of CCL4 on nCM mitosis and cytokinesis. G-H) Immunostaining images and quantification of Ki67+ HUVEC. Scale bar = 50 µm (**p<0.01; ***p<0.001; ns: not significant, one-way ANOVA with Šídák's multiple comparisons test).

Supplementary Material 4: Figure S4. Pool3 reduces the apoptosis rate and enhances angiogenesis of HUVEC in vitro. A-B) Immunofluorescence images and quantification of cCasp3+ HUVEC (B). C) Bright-field (BF) images of the scratch assay using HUVEC. The red line indicates the initial scratched area, and the black line shows the area after 15 h in the Ctrl and Pool3. D) Quantification of the migration rate in the scratch assay. E) BF images of tube formation of HUVEC in different conditions. F-G) Quantification of the average number of branching points and closed tubes formed by HUVEC in the mentioned conditions. Scale bar in A = 100 µm, C = 400 µm, and E = 200 µm (*p<0.05; **p<0.01; ***p<0.001; ns: not significant, one-way ANOVA with Šídák's multiple comparisons test in B, F, and G, unpaired t-test in D).

Supplementary Material 5: Figure S5. Pool3-treated anillin transgenic nCM confirmed enhanced cytokinesis rate. A) Immunostaining images showing single channels for Ki67 and PHH3 staining in Pool3 and Ctrl-treated nCM. B) Schematic image of the experiments. C) Schematic image showing the location of anillin signal in nCM from αMHC-H2BmCherry x CAG-eGFP-Anillin transgenic mouse heart indicates different stages of cell cycle activity (Figure is taken from M. Hesse et al. Nat Commun, 2012)26. D-E) Quantification of anillin signal in nCM cytoplasm indicating nCM in M phase (D), in the nuclei showing cycling nCM (E), between two nuclei, showing binucleation (F). G) Immunostaining images showing single channels for cCasp3 and TUNEL staining in Pool3 and Ctrl-treated nCM. Scale bar = 100 µm (ns: non-significant; one-way ANOVA with Šídák's multiple comparisons test).

Supplementary Material 6: Figure S6. STRING analysis showing protein-protein interactions of Pool3 with TLR2 in the treated nCM and 1 dps P1 MI. A-B) STRING analysis of upregulated genes in Pool3-treated nCM and k-means clustering with related genes in each cluster (B). C) Immunostaining for BCL2 in Ctrl, Pool3, and zymosan-treated nCM. Scale bar = 50 µm.

Supplementary Material 7: Figure S7. Tlr2, as the key hub, is upregulated after P1 nTAC, MI, and in vitro upon Pool3 treatment. A-B) DotPlot showing survival and cell-cycle-associated genes enriched in Tlr2-positive compared with Tlr2-negative nTAC nCM within the regenerative/cycling CM2 subpopulation. Dot size indicates the fraction of expressing cells, while color represents average expression. C) Venn diagram showing the overlap in upregulated genes in 1 dps P1 MI and Pool3-treated nCM. D) STRING analysis showing protein-protein interaction and k-means clustering of overlapping upregulated genes 1 dps P1 MI and Pool3-treated nCM. E) Gene list related to each cluster identified by k-means clustering.

Supplementary Material 8: Figure S8. TLR2 KO nCM exhibit a similar cell cycle activity rate, yet reduced baseline apoptosis compared to wild-type CD1 nCM in vitro, and TLR2 is overexpressed in nCM after MI and nTAC, but is downregulated with age. A-E) Comparison of cell cycle activity and apoptosis rates of TLR2 KO and CD1 wild-type nCM in vitro in Ctrl and Pool3 treatments. F) Gene expression levels of Tlr 1, 2, 6 in nCM from Ctrl and 14 dps P0 nTAC hearts analyzed from publicly available single-nucleus RNA seq data27. G) Tlr2 expression in nCM in P1 and P56 sham and 2 dps MI from publicly available database21. H-I) Western blot and quantification of TLR2 in the LV of P14 n.i. and 14 dps P0 nTAC hearts. (*p<0.05; **p<0.01; ***p<0.001; ns: not significant; one-way ANOVA with Šídák's multiple comparisons test in A-E and G; unpaired t-test in I).

Supplementary Material 9: Figure S9. Expression of TLR2 increases upon nTAC but reduces with age. A) Immunostaining images of TLR2 staining in heart tissue sections at P14 after P0 nTAC and n.i. as well as adult and P3 n.i. hearts. B) Immunostaining images showing a single channel of Ki67 and PHH3 in Z-488 and Ctrl-treated nCM. C) Immunostaining images showing a single channel for cCasp3 and TUNEL in Z-488 and Ctrl-treated nCM. Scale bar in A = 50 µm, in B, C = 20 µm

Supplementary Material 10: Figure S10. Uncropped Western blot images used for protein expression analysis. Uncropped original Western blot images, including the molecular weight ladder, used to assess protein expression in the LV of P14 non-injured (n.i.) hearts and hearts collected 14 days after P0 nTAC. A–B) Single-channel images showing BCL2 (A) and TLR2 (B), together with the corresponding GAPDH loading-control images and merged images.

## Data Availability

All new sequencing data obtained in this study are deposited in the Short Read Archive at the National Center for Biotechnology Information under the BioProject ID: PRJNA1070843.
